# Evaluation of comorbidity measures for predicting mortality and revision surgery after elective primary shoulder replacement surgery based on data from the National Joint Registry and Hospital Episode Statistics for England: population based cohort study

**DOI:** 10.1136/bmjmed-2024-001283

**Published:** 2025-08-10

**Authors:** Epaminondas Markos Valsamis, Adrian Sayers, Jie Ma, Paula Dhiman, Stephen E Gwilym, Jonathan L Rees

**Affiliations:** 1 Nuffield Department of Orthopaedics, Rheumatology and Musculoskeletal Sciences, University of Oxford, Oxford, UK; 2 NIHR Oxford Biomedical Research Centre, Oxford, UK; 3 Muscloskeletal Research Unit, University of Bristol, Bristol, UK; 4 Centre for Statistics in Medicine, Nuffield Department of Orthopaedics, Rheumatology and Musculoskeletal Sciences, University of Oxford, Oxford, UK

**Keywords:** Health policy, Musculoskeletal diseases, Orthopedics, Upper extremity

## Abstract

**Objective:**

To determine the importance of comorbidity measures when predicting mortality and revision surgery after elective primary shoulder replacement surgery.

**Design:**

Population based cohort study.

**Setting:**

Linked data from the National Joint Registry and NHS Hospital Episode Statistics were used to identify all elective primary shoulder replacements in England, 6 January 2012 to 30 March 2022.

**Participants:**

37 176 consenting patients, aged 18-100 years, who had elective primary shoulder replacement surgery.

**Main outcome measures:**

Risk of mortality at 90 and 365 days, and risk of long term revision surgery after the primary surgery.

**Results:**

37 176 primary shoulder replacement procedures were included; 102 patients died within 90 days and 445 within 365 days of the primary surgery. 1219 patients had revision surgery over a maximum follow-up period of >10 years. The addition of comorbidity measures derived from Hospital Episode Statistics (Charlson comorbidity index with summary hospital mortality index weights, Elixhauser comorbidity index, and hospital frailty risk score) to simpler models resulted in little improvement in predictive performance. Optimism adjusted performance (C index) of the models that included age, sex, American Society of Anesthesiologists (ASA) grade, and main surgical indication was 0.76 (95% confidence interval (CI) 0.72 to 0.81) for 90 day mortality, 0.74 (0.71 to 0.76) for 365 day mortality, and 0.64 (0.63 to 0.66) for revision surgery. The best performing models that included a comorbidity measure had an optimism adjusted C index of 0.77 (95% CI 0.73 to 0.81) for 90 day mortality, 0.76 (0.74 to 0.78) for 365 day mortality, and 0.65 (0.63 to 0.66) for revision surgery. Heterogeneity in model performance across regions of England was low, and decision curve analysis showed minimal improvement in net benefit when including comorbidity measures.

**Conclusions:**

In this study, patient comorbidity scores added little improvement to simpler models that included age, sex, ASA grade, and main surgical indication for predicting mortality and revision surgery after elective primary shoulder replacement surgery. This improvement needs to be balanced against the additional challenges of routine data linkage to obtain these scores.

**WHAT IS ALREADY KNOWN ON THIS TOPIC**

- The comparative value of including comorbidity measures to predict mortality or revision surgery after shoulder replacement surgery has not been investigated
- Identifying the most important predictors of mortality and revision surgery after shoulder replacement surgery is needed for outlier analysis by national joint registries

**WHAT THIS STUDY ADDS**

- Addition of comorbidity scores from other databases provided little improvement in model performance over simpler models that included age, sex, American Society of Anesthesiologists (ASA) grade, and main surgical indication for predicting mortality at 90 and 365 days, and long term revision surgery, after elective primary shoulder replacement surgery
- The improvement in model performance needs to be balanced against the additional burden and logistic complexity of routine data linkage to derive comorbidity scores

**HOW THIS STUDY MIGHT AFFECT RESEARCH, PRACTICE, OR POLICY**

- The patient and surgeon factors identified should be used for case mix adjustment during outlier analysis for surgeons and hospitals undertaking primary elective shoulder replacement surgery
- When considering data linkage to obtain comorbidity scores, decision makers should weigh the small performance gain against the practical challenges of implementation

## Introduction

 Shoulder replacement is an effective treatment option for end stage glenohumeral arthritis, and the incidence of this surgery is increasing internationally, with >8000 primary shoulder replacement procedures undertaken in the UK each year.^1^,^4^ Recent findings, however, have shown that the risk of serious adverse events after shoulder replacement surgery is higher than previously thought.^5^ Monitoring joint replacement outcomes is important at the implant, surgeon, and hospital levels, to maintain and improve the quality of healthcare delivered to patients, with an increasing number of national joint registries using outlier analysis methods to support this monitoring.^6^,^8^ Variations in performance between surgeons and hospitals might be because of differences in patient and surgical characteristics. For example, some surgeons or hospitals might treat patients at higher risk or perform more complex surgical procedures. Hence case mix adjustment for these differences must form an integral part of outlier analysis.^6^

Selecting the most appropriate variables for case mix adjustment means understanding their predictive value and availability in routinely collected datasets. For example, the presence of comorbidities is known to be associated with worse health outcomes, but these conditions are not routinely collected by national joint registries, and obtaining these data usually requires linkage to hospital datasets.^9 10^ The National Joint Registry for England, Wales, Northern Ireland, the Isle of Man, and the States of Guernsey already undertakes successful outlier analysis for hip and knee replacement surgeries at the surgeon and hospital levels based on patient mortality and revision surgery, but this analysis is not being done for shoulder replacement surgery.^7^ The aim of this study was to determine the importance of comorbidity measures when predicting mortality and revision surgery to inform case mix adjustment in outlier analysis for elective primary shoulder replacement surgery.

## Methods

### Study design and data sources

This population based prospective cohort study used linked data from the National Joint Registry and NHS Hospital Episode Statistics database. All primary (first) elective shoulder replacements performed at public hospitals, and publicly funded procedures in private hospitals in England were identified from 6 January 2012 to 30 March 2022. Data submission to the National Joint Registry is mandatory for all public and private hospitals, and information on patients, surgeons, and operations is collected. The NHS Hospital Episode Statistics Admitted Patient Care database records all inpatient and day case activity in public hospitals, and publicly funded procedures at private hospitals in England and records demographic data, medical diagnoses, and procedural and administrative information. Hospital Episode Statistics data are used for accurate reimbursement of NHS providers for their activities. A sample size of convenience was used for the analysis and was based on the linked data available from the National Joint Registry and Hospital Episode Statistics for our study period.

### Participants

All consenting patients aged 18-100 years having elective primary shoulder replacement surgery were eligible for inclusion in the study. Data for sex were taken from information in the National Joint Registry. All three main types of shoulder replacement (ie, humeral hemiarthroplasty, anatomical total shoulder replacement, and reverse total shoulder replacement) were included. Patients were included if their surgical history was consistent (ie, date of death did not precede their surgery) and did not contain duplicate data. Patients having surgery for acute trauma or malignancy were excluded. The unit of analysis was considered the procedure rather than the patient.

### Outcomes

The outcomes of interest were mortality and revision surgery. The National Joint Registry defines revision as a procedure that involves adding, removing, or modifying one or more components of a joint prosthesis. Data on mortality were from the NHS England National Back Office Tracing Service, which was linked to data from the National Joint Registry, and revision procedures were recorded by the National Joint Registry.^11^ Mortality was studied at 90 days and 365 days after surgery, to capture the initial short term increase in mortality from the surgical procedure, in keeping with previous analyses on hip and knee replacement surgery.^10^ Revision surgery was studied over a 10.1 year follow-up period because we were interested in long term implant survival. The National Joint Registry currently undertakes revision surgery outlier analysis for hip and knee replacements based on five and 10 year time periods. Patients were censored at the end of the study period and data were reformatted to include time to event variables in a format suitable for survival analysis.

### Statistical analysis

Variables included in the models were chosen based on those routinely collected by national joint registries, as well as considering some of the more established comorbidity measures that usually require linkage to hospital databases. Patient age was modelled with restricted cubic splines, with three knots to allow for clinically plausible non-linearity. Restricted cubic splines can capture complex non-linear relationships while ensuring smoothness. Age, sex, and the American Society of Anesthesiologists (ASA) grade (categorical: I, II, III, IV and V) were the covariates for our reference model. More complex models included the addition of the main indication for surgery (categorical: osteoarthritis, cuff tear arthropathy, inflammatory arthropathy, avascular necrosis, trauma sequelae, and other), the index of multiple deprivation tenth (categorical, based on patients’ lower layer super output area), and one of three comorbidity scores. No variable selection techniques were used during the modelling itself. Comorbidity scores were the Charlson comorbidity index with summary hospital mortality index weights, Elixhauser comorbidity index, and hospital frailty risk score, and were determined with ICD-10 (international classification of diseases, 10th revision) codes from Hospital Episode Statistics data for hospital episodes starting at or before the date of the primary procedure. The Charlson comorbidity index with summary hospital mortality index weights was a categorical variable (0, 1-5, and >5) and is based on the clinical conditions identified in the Charlson comorbidity index, adapted for a UK based outcome system.^12^ The van Walraven modification of the Elixhauser comorbidity score was a continuous variable, and after testing for non-linearity, was modelled as linear for mortality and with restricted cubic splines for revision surgery.^13^ The hospital frailty risk score was a categorical variable (<5, 5-15, and >15).^14^

Flexible parametric survival models (with restricted cubic splines to allow for modelling non-linearity in the baseline hazard function) were used to predict the risk of mortality at each time point, and revision surgery, for the reference model and for all of the more complex models that used additional predictor variables. Separate models were developed for mortality by censoring patients at each time point (90 and 365 days). The proportional hazards assumption was checked for all covariates by graphical inspection, and no clear violations were identified.

Calibration is a measure of the accuracy of risk estimates and relates to the agreement between the estimated and observed event rate.^15^ Model calibration was assessed graphically by calibration plots at specific time points (90 and 365 days for mortality, and five years for revision surgery because only a small number of patients had 10 year follow-up data) and quantified with the calibration slope calculated across all time points for each model. Discrimination, a measure of the model's ability to discriminate between individuals who have and do not have the event, was assessed with the C index. Internal validation with bootstrapping (with 200 bootstrap samples) was used to assess model overfitting. Harrell's bias correction was used: in this approach, a prediction model is constructed for each bootstrap sample and its performance is assessed on both the bootstrap sample and the original population. The bootstrap estimate of optimism is then calculated by averaging the difference between bootstrap model performance and original population model performance across all bootstrap samples, and confidence intervals (CIs) were calculated with the location shifted approach.^16 17^ Shrinkage was applied to the original model coefficients using the optimism adjusted calibration slope, and the model intercept was re-estimated to ensure calibration in the large.

Apparent and optimism adjusted model performance were reported. Model optimism refers to the tendency of a prediction model to perform better on the dataset on which it was developed than on new data, often because of overestimating its true predictive ability. The clinical utility of each model was assessed with decision curve analysis. Decision curve analysis reports the net benefit of using the model to decide who to treat over alternative strategies, specifically treating all patients regardless of their risk, or not treating any patient to avoid any risk.^18^ The net benefit is the difference between the proportion of true positives and false positives, weighted on the threshold (willingness to forego surgery to avoid mortality or revision surgery) for designating a patient as high risk. A model is considered to have clinical utility if it has net benefit over the range of risk thresholds that would designate a patient as high risk.^19^ In this exploratory study, we did not establish a risk threshold of interest a priori, but were interested in the relative predictive performance of each model when assessed with decision curve analysis.

Internal-external cross validation was performed to examine heterogeneity between regions and assess generalisability across the regions of treatment included in the National Joint Registry. For this cross validation, each of the 10 geographical regions were iteratively excluded from the development dataset, and the models were developed on the remaining regions and validated on the omitted region. Models were not adjusted for overfitting. We used a random effects meta-analysis with the DerSimonian-Laird estimator to calculate the pooled C index and calibration slope across regions, and forest plots were examined to assess heterogeneity.

We excluded 384 procedures with a missing value for index of multiple deprivation, representing 1.0% of all procedures, and performed a complete case analysis.^20 21^ Data were not missing for any other variable or outcome used in the analysis. All analyses were done with Stata version 18.5 (StataCorp).^22^

### Patient and public involvement

Two of the top 10 research uncertainties from the 2015 James Lind Alliance Priority Setting Partnership on shoulder surgery related to predicting patient outcomes after surgery and accessing appropriate healthcare pathways and services.^23^ Patient representatives sit on the committee structure of the National Joint Registry where outlier analysis is considered to be an important assessment in ensuring best outcomes for patients. 

The National Joint Registry and its patient representative group have been made aware of the results of this study and will support the dissemination of results through their networks. Dissemination to the wider public will be supported by the University of Oxford and the Oxford National Institute for Health and Care Research Biomedical Research Centre through the press, media, and charities. Surgeons and policy makers will be informed of the results through presentations at national and international scientific meetings.

## Results

### Patient characteristics

A total of 37 176 shoulder replacement procedures met the eligibility criteria for the study; 102 patients died within 90 days of the primary surgery (3 296 205 days of total analysis time), 445 died within 365 days of the primary surgery (12 782 965 days of total analysis time), and 1219 had revision surgery within 10.1 years of the primary surgery (159 722 years of total analysis time). At both time points, patients who died were, on average, more likely to be male, older, have a high ASA grade, have surgery for cuff tear arthropathy or for trauma sequelae, and have higher comorbidity scores (table 1). Patients who had revision surgery were, on average, more likely to be male, younger, have surgery for trauma sequelae, and have a humeral hemiarthroplasty.

**Table 1:** Baseline characteristics for the study population by 90 day and 365 day mortality, and by revision surgery status

	90 day mortality		365 day mortality		Revision surgery	
	Alive (n=37 074)	Dead (n=102)	Alive (n=36 731)	Dead (n=445)	No (n=35 957)	Yes (n=1219)
Mean (SD) age (years)	72.0 (9.7)	76.8 (9.1)	72.0 (9.7)	76.5 (9.7)	72.2 (9.6)	67.2 (11.1)
Sex:						
Male	11 213 (30.2)	39 (38.2)	11 076 (30.2)	176 (39.6)	10 747 (29.9)	505 (41.4)
Female	25 861 (69.8)	63 (61.8)	25 655 (69.8)	269 (60.4)	25 210 (70.1)	714 (58.6)
ASA grade:						
1	2298 (6.2)	1 (1.0)	2292 (6.2)	7 (1.6)	2181 (6.1)	118 (9.7)
2	23 508 (63.4)	34 (33.3)	23 369 (63.6)	173 (38.9)	22 770 (63.3)	772 (63.3)
3	10 980 (29.6)	59 (57.8)	10 796 (29.4)	243 (54.6)	10 724 (29.8)	315 (25.8)
4 and 5	288 (0.8)	8 (7.8)	274 (0.7)	22 (4.9)	282 (0.8)	14 (1.1)
Main surgical indication:						
Avascular necrosis	925 (2.5)	1 (1.0)	914 (2.5)	12 (2.7)	882 (2.5)	44 (3.6)
Cuff tear arthropathy	10 399 (28.0)	38 (37.3)	10 289 (28.0)	148 (33.3)	10 181 (28.3)	256 (21.0)
Inflammatory arthropathy	1439 (3.9)	0 (0.0)	1421 (3.9)	18 (4.0)	1380 (3.8)	59 (4.8)
Osteoarthritis	20 642 (55.7)	39 (38.2)	20 502 (55.8)	179 (40.2)	20 019 (55.7)	662 (54.3)
Other	1101 (3.0)	8 (7.8)	1086 (3.0)	23 (5.2)	1072 (3.0)	37 (3.0)
Trauma sequelae	2568 (6.9)	16 (15.7)	2519 (6.9)	65 (14.6)	2423 (6.7)	161 (13.2)
Index of multiple deprivation tenth:						
1 (most deprived)	2719 (7.3)	9 (8.8)	2689 (7.3)	39 (8.8)	2611 (7.3)	117 (9.6)
2	2634 (7.1)	9 (8.8)	2609 (7.1)	34 (7.6)	2556 (7.1)	87 (7.1)
3	2903 (7.8)	8 (7.8)	2875 (7.8)	36 (8.1)	2810 (7.8)	101 (8.3)
4	3564 (9.6)	11 (10.8)	3523 (9.6)	52 (11.7)	3446 (9.6)	129 (10.6)
5	3864 (10.4)	8 (7.8)	3822 (10.4)	50 (11.2)	3756 (10.4)	116 (9.5)
6	4343 (11.7)	10 (9.8)	4306 (11.7)	47 (10.6)	4224 (11.7)	129 (10.6)
7	4480 (12.1)	18 (17.6)	4448 (12.1)	50 (11.2)	4375 (12.2)	123 (10.1)
8	4336 (11.7)	14 (13.7)	4297 (11.7)	53 (11.9)	4220 (11.7)	130 (10.7)
9	4249 (11.5)	9 (8.8)	4211 (11.5)	47 (10.6)	4093 (11.4)	165 (13.5)
10 (least deprived)	3982 (10.7)	6 (5.9)	3951 (10.8)	37 (8.3)	3866 (10.8)	122 (10.0)
Summary hospital mortality index:						
1	13 773 (37.2)	14 (13.7)	13 722 (37.4)	65 (14.6)	13 296 (37.0)	491 (40.3)
2	8107 (21.9)	14 (13.7)	8061 (21.9)	60 (13.5)	7867 (21.9)	254 (20.8)
3	15 194 (41.0)	74 (72.5)	14 948 (40.7)	320 (71.9)	14 794 (41.1)	474 (38.9)
Mean (SD) Elixhauser comorbidity score	2.7 (6.0)	7.9 (8.6)	2.7 (6.0)	7.8 (8.4)	2.7 (6.0)	2.6 (6.2)
Hospital frailty risk score:						
1	17 916 (48.3)	21 (20.6)	17 821 (48.5)	116 (26.1)	17 364 (48.3)	573 (47.0)
2	15 449 (41.7)	46 (45.1)	15 307 (41.7)	188 (42.2)	14 985 (41.7)	510 (41.8)
3	3709 (10.0)	35 (34.3)	3603 (9.8)	141 (31.7)	3608 (10.0)	136 (11.2)
Primary procedure type:						
Humeral hemiarthroplasty	4815 (13.0)	8 (7.8)	4773 (13.0)	50 (11.2)	4498 (12.5)	325 (26.7)
Reverse total shoulder replacement	20 055 (54.1)	78 (76.5)	19 821 (54.0)	312 (70.1)	19 650 (54.6)	483 (39.6)
Anatomical total shoulder replacement	12 204 (32.9)	16 (15.7)	12 137 (33.0)	83 (18.7)	11 809 (32.8)	411 (33.7)

Data are number (%) unless indicated otherwise.

ASA, American Society of Anesthesiologists; SD, standard deviation.

### Models

Six incrementally more complex models were used to predict 90 day and 365 day mortality as well as long term revision surgery (table 2). Although more complex models showed improved apparent discrimination, on internal validation (with bootstrapping) their optimism adjusted performance was only slightly superior. Model optimism was higher for 90 day mortality models than for 365 day mortality models. Model optimism was higher for more complex models. Model optimism was the lowest for revision surgery models. The optimism adjusted C index for 90 day mortality models ranged from 0.74 to 0.77 (where a value of 1 would represent perfect discrimination), with the reference model having the lowest discrimination and model 6 (when the hospital frailty risk score was included) having the highest discrimination.

The optimism adjusted C index for the 365 day mortality models ranged from 0.72 to 0.76, with the reference model having the lowest discrimination and models 4-6 (with any of the three comorbidity scores included) having the highest discrimination. Among mortality models for both time points, increasing model complexity was generally associated with a reduction in the optimism adjusted calibration slope (where a value of 1 would represent perfect alignment of predicted probabilities with observed outcomes), indicating greater overfitting.

**Figure 1:**
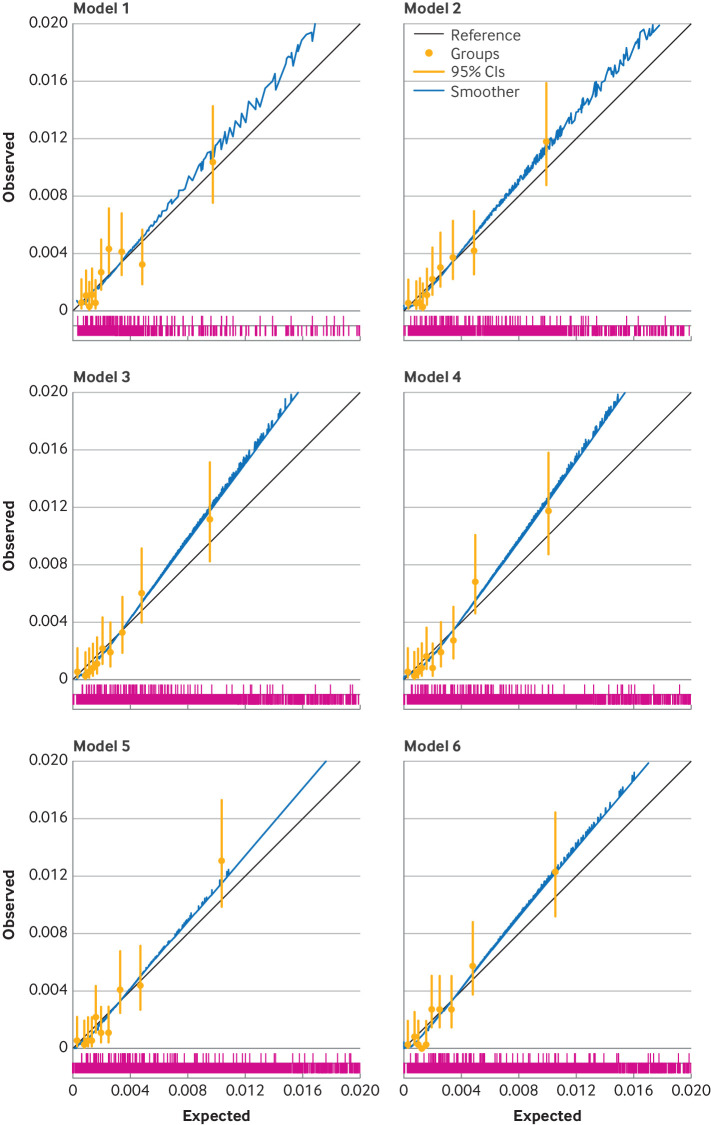
Calibration plots for six incrementally more complex models predicting 90 day mortality (optimism adjusted model fit). Model 1 is the reference model, with covariates age, sex, and American Society of Anesthesiologists (ASA) grade; model 2=model 1+main surgical indication; model 3=model 2+index of multiple deprivation tenth; model 4=model 3+Charlson comorbidity index with summary hospital mortality index score; model 5=model 3+Elixhauser comorbidity score; and model 6=model 3+hospital frailty risk score. The spike plot of the distribution of events and non-events is shown in pink. CI=confidence interval

The optimism adjusted C index for revision surgery was lower than for the mortality models and ranged from 0.63 to 0.65, with the reference model having the lowest discrimination and model 6 having a marginally higher discrimination than the rest of the models. Figures1^3^ show calibration plots of the optimism adjusted fit of the models for mortality at 90 days and 365 days, and for revision surgery (at five years).

**Figure 2:**
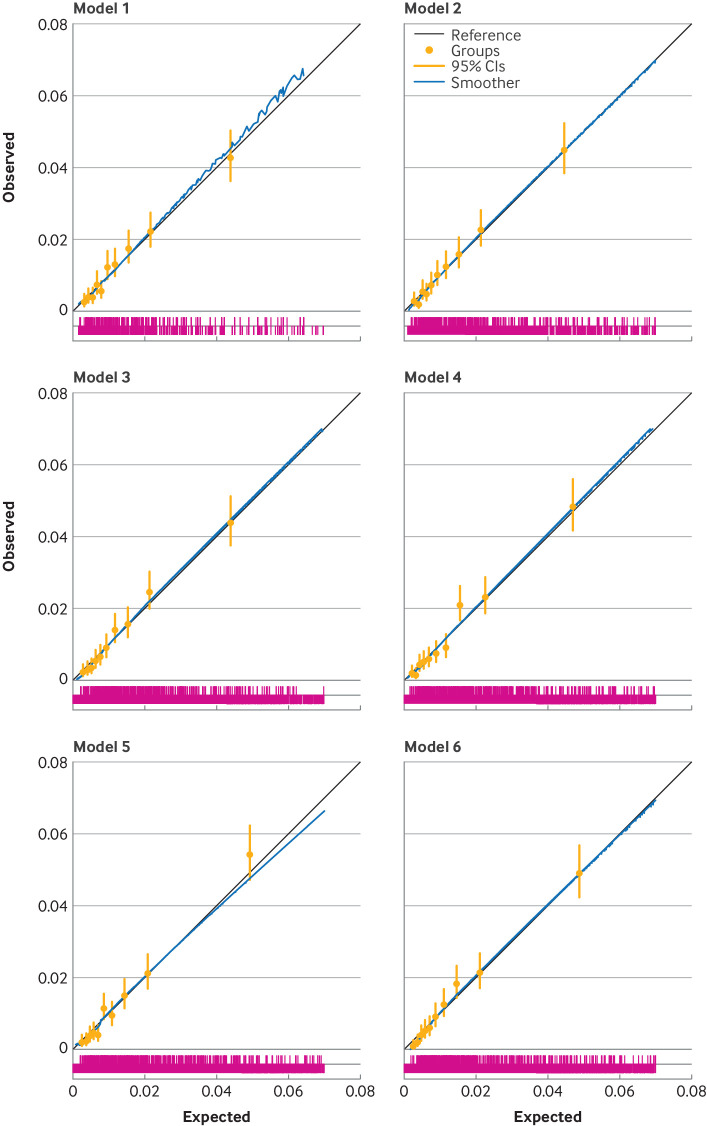
Calibration plots for six incrementally more complex models predicting 365 day mortality (optimism adjusted model fit). Model 1 is the reference model, with covariates age, sex, and American Society of Anesthesiologists (ASA) grade; model 2=model 1+main surgical indication; model 3=model 2+index of multiple deprivation; model 4=model 3+Charlson comorbidity index with summary hospital mortality index score; model 5=model 3+Elixhauser comorbidity score; and model 6=model 3+hospital frailty risk score. The spike plot of the distribution of events and non-events is shown in pink. CI=confidence interval.

**Figure 3:**
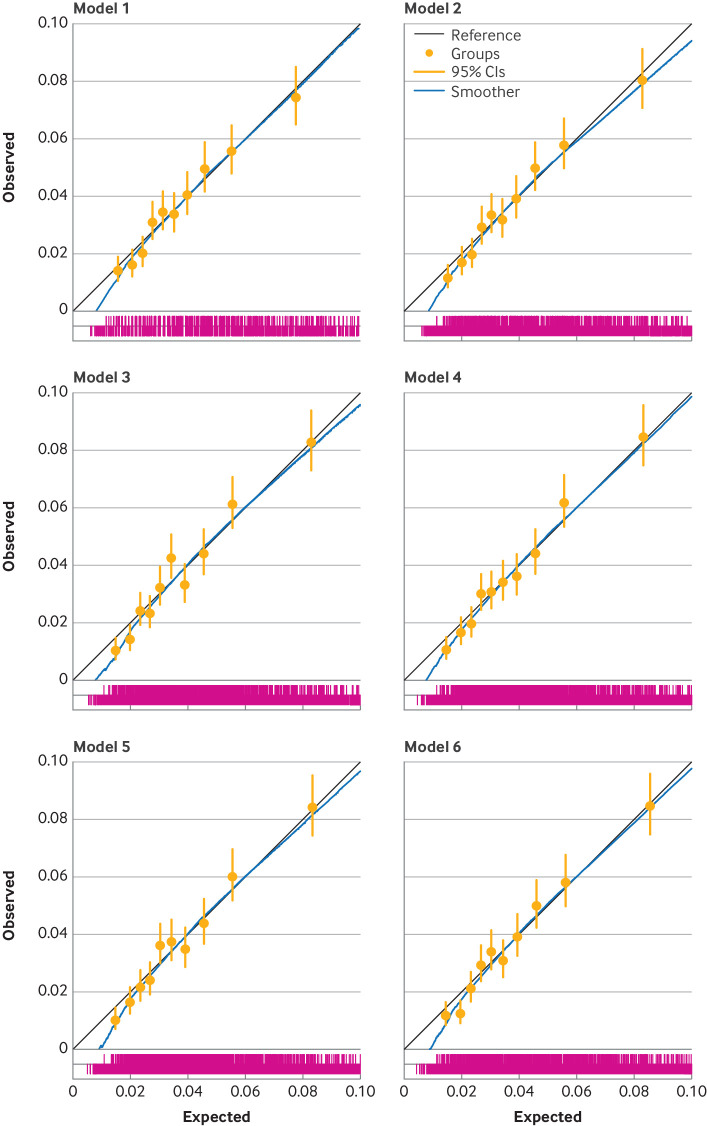
Calibration plots for six incrementally more complex models predicting revision surgery at five years (optimism adjusted model fit). Model 1 is the reference model, with covariates age, sex, and American Society of Anesthesiologists (ASA) grade; model 2=model 1+main surgical indication; model 3=model 2+index of multiple deprivation tenth; model 4=model 3+Charlson comorbidity index with summary hospital mortality index score; model 5=model 3+Elixhauser comorbidity score; and model 6=model 3+hospital frailty risk score. The spike plot of the distribution of events and non-events is shown in pink. CI=confidence interval.

**Table 2:** Apparent and optimism adjusted (bootstrap internal validation) model performance for all models, for 90 day and 365 day mortality, and revision surgery

Model No	Model description	Apparent performance (estimate (95% CI))		Optimism adjusted performance (estimate (95% CI))	
		C index	Calibration slope	C index	Calibration slope
90 day mortality					
1	Reference (age, sex, and ASA grade)	0.75 (0.70 to 0.79)	1.00 (0.81 to 1.19)	0.74 (0.69 to 0.79)	0.92 (0.73 to 1.11)
2	Model 1+main surgical indication	0.78 (0.73 to 0.82)	1.00 (0.83 to 1.18)	0.76 (0.72 to 0.81)	0.86 (0.68 to 1.03)
3	Model 2+index of multiple deprivation tenth	0.79 (0.74 to 0.83)	1.00 (0.83 to 1.17)	0.75 (0.71 to 0.80)	0.80 (0.63 to 0.97)
4	Model 3+Charlson comorbidity index with summary hospital mortality index score	0.80 (0.75 to 0.84)	1.00 (0.83 to 1.17)	0.76 (0.72 to 0.81)	0.80 (0.63 to 0.96)
5	Model 3+Elixhauser comorbidity score	0.80 (0.75 to 0.84)	1.00 (0.85 to 1.15)	0.76 (0.72 to 0.81)	0.82 (0.67 to 0.98)
6	Model 3+hospital frailty risk score score	0.81 (0.76 to 0.85)	1.00 (0.84 to 1.16)	0.77 (0.73 to 0.81)	0.81 (0.65 to 0.97)
365 day mortality					
1	Reference (age, sex, and ASA grade)	0.73 (0.70 to 0.75)	1.00 (0.90 to 1.10)	0.72 (0.70 to 0.75)	0.97 (0.87 to 1.07)
2	Model 1+main surgical indication	0.74 (0.72 to 0.77)	1.00 (0.90 to 1.10)	0.74 (0.71 to 0.76)	0.96 (0.87 to 1.06)
3	Model 2+index of multiple deprivation tenth	0.75 (0.73 to 0.77)	1.00 (0.91 to 1.09)	0.74 (0.71 to 0.76)	0.94 (0.85 to 1.04)
4	Model 3+Charlson comorbidity index with summary hospital mortality index score	0.77 (0.75 to 0.79)	1.00 (0.91 to 1.09)	0.76 (0.74 to 0.78)	0.94 (0.85 to 1.03)
5	Model 3+Elixhauser comorbidity score	0.77 (0.75 to 0.80)	1.00 (0.92 to 1.08)	0.76 (0.74 to 0.79)	0.95 (0.87 to 1.03)
6	Model 3+hospital frailty risk score	0.77 (0.75 to 0.79)	1.00 (0.91 to 1.09)	0.76 (0.74 to 0.78)	0.95 (0.86 to 1.03)
Revision surgery					
1	Reference (age, sex, and ASA grade)	0.63 (0.61 to 0.64)	1.00 (0.88 to 1.12)	0.63 (0.61 to 0.64)	0.97 (0.86 to 1.09)
2	Model 1+main surgical indication	0.65 (0.63 to 0.66)	1.00 (0.90 to 1.10)	0.64 (0.63 to 0.66)	0.97 (0.86 to 1.07)
3	Model 2+index of multiple deprivation tenth	0.65 (0.63 to 0.66)	1.00 (0.90 to 1.10)	0.64 (0.63 to 0.66)	0.95 (0.84 to 1.05)
4	Model 3+Charlson comorbidity index with summary hospital mortality index score	0.65 (0.63 to 0.67)	1.00 (0.90 to 1.10)	0.64 (0.63 to 0.66)	0.94 (0.84 to 1.04)
5	Model 3+Elixhauser comorbidity score	0.65 (0.63 to 0.67)	1.00 (0.90 to 1.10)	0.64 (0.63 to 0.66)	0.94 (0.84 to 1.04)
6	Model 3+hospital frailty risk score	0.65 (0.64 to 0.67)	1.00 (0.90 to 1.10)	0.65 (0.63 to 0.66)	0.94 (0.85 to 1.04)

Model optimism for each performance metric is represented by the difference between the optimism adjusted model performance and the apparent model performance.

ASA, American Society of Anesthesiologists; CI, confidence interval.

Internal-external cross validation was only possible for 365 day mortality and revision surgery because of model convergence issuesas a result of the small event rate for mortality at 90 days. We found that heterogeneity in model performance across different geographical regions was low for all models for each outcome (online supplemental material).

### Decision curve analysis

**Figure 4:**
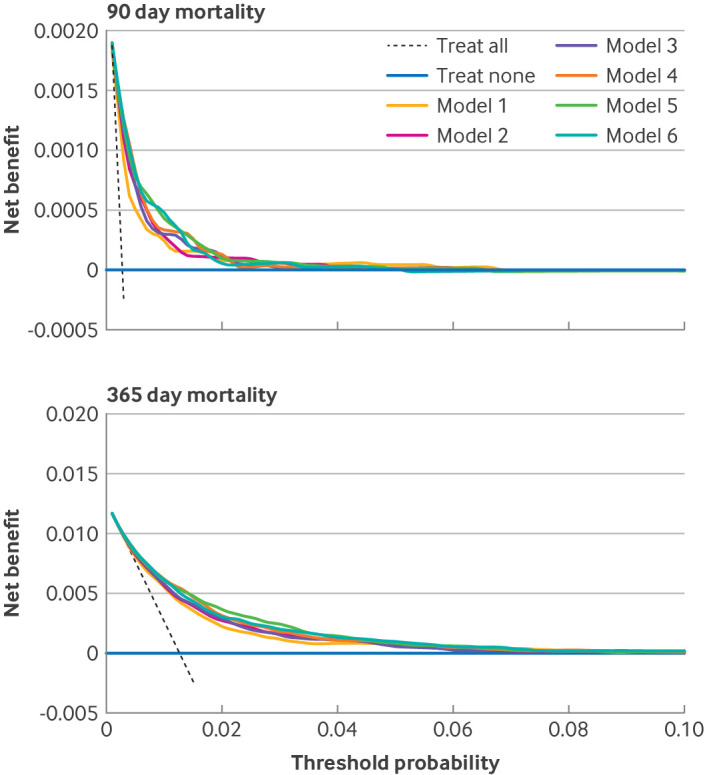
Decision curve analysis for six incrementally more complex models predicting 90 day (top) and 365 day (bottom) mortality (optimism adjusted model fit). Model 1 is the reference model, with covariates age, sex, and American Society of Anesthesiologists (ASA) grade; model 2=model 1+main surgical indication; model 3=model 2+index of multiple deprivation tenth; model 4=model 3+Charlson comorbidity index with summary hospital mortality index score; model 5=model 3+Elixhauser comorbidity score; and model 6=model 3+hospital frailty risk score

**Figure 5:**
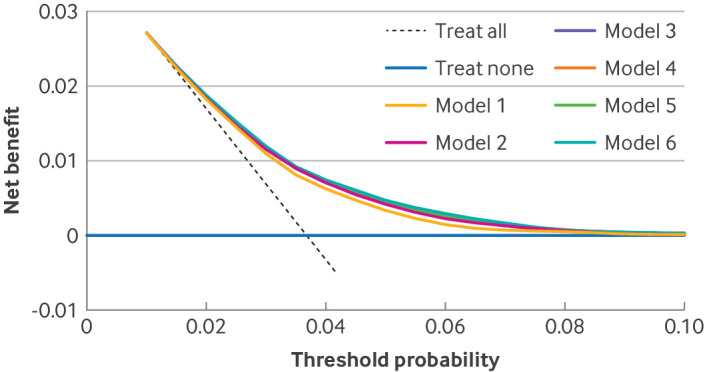
Decision curve analysis for six incrementally more complex models predicting revision surgery at five years (optimism adjusted model fit). Model 1 is the reference model, with covariates age, sex, and American Society of Anesthesiologists (ASA) grade; model 2=model 1+main surgical indication; model 3=model 2+index of multiple deprivation tenth; model 4=model 3+Charlson comorbidity index with summary hospital mortality index score; model 5=model 3+Elixhauser comorbidity score; and model 6=model 3+hospital frailty risk score

Decision curve analysis of the optimism adjusted fit of the models showed net benefit for all models predicting 90 day mortality up to a risk threshold of about 3%, with minimal deviation into negative values (ie, that suggest net harm) at some higher risk thresholds (figure 4). All models predicting 365 day mortality (figure 4) and revision surgery at five years (figure 5) had net benefit at all of the risk thresholds examined. For mortality, different models had comparatively greater net benefit at different risk thresholds, meaning that no one model was identified as superior based on decision curve analysis. For revision surgery, all models more complex than the reference model seemed to have net benefit compared with the reference model at all of the risk thresholds examined.

## Discussion

### Principal findings

In this study, we used prospectively collected linked data from the National Joint Registry and NHS Hospital Episode Statistics for England to identify the importance of comorbidity measures for predicting mortality and revision surgery after primary elective shoulder replacement surgery to inform case mix adjustment for outlier analysis. We found that a model with age, sex, ASA grade, and main surgical indication provided good and moderate predictive performance for mortality and revision surgery, respectively. More complex models with more variables, including comorbidity scores, offered little improvement in model performance for either outcome. Deriving comorbidity scores can be challenging because linkage of national joint registry data to other hospital datasets is required, an approach with added burden, logistical difficulties, and potential coding errors. Given these challenges, the need for routine data linkage to obtain comorbidity variables for predicting mortality at 90 and 365 days or long term revision surgery after elective primary shoulder replacement surgery needs to be balanced against the small improvement in model performance.

### Comparison with other studies

To our knowledge, the comparative value of including comorbidity measures to predict mortality or revision surgery in shoulder replacement surgery has not been investigated. A few studies have assessed predictors of mortality or revision surgery by reporting the P values of a multivariable model that included relevant predictor variables. P values (or associated hazard ratios) provide no information about how well a model is calibrated and can be misleading.^24^ A small cohort study (n=640) by Dacombe and colleagues, from one centre, investigated predictors of mortality at 30, 90, and 365 days after primary and revision shoulder replacement surgery for elective and acute trauma indications.^25^ A small number of deaths were reported and the authors did not test incrementally more complex models, but a significant association was found between mortality and medical comorbidities and surgical indication. Their small sample size, however, might explain why other variables, such as age and sex, were not found to be significantly associated with mortality.

One larger study from the US of 4019 patients having primary shoulder replacement surgery for elective and acute trauma indications identified that comorbidities and ASA grade were associated with mortality.^26^ A recent study with a large sample size (n=108 667) investigated predictors of longer term mortality after surgery for both elective and acute trauma indications, and identified that age, sex, surgical indication, and medical comorbidities were significantly associated with five year mortality, but their analysis was also restricted to reporting P values from one multivariate model.^27^ A cohort study from the US identified that age, sex, body mass index, comorbidity, ASA grade, and underlying diagnosis were predictive factors of revision surgery after total shoulder replacement.^28^ Like other studies, however, only P values from multivariable models were reported.

One study investigating predictors of mortality after primary elective hip and knee replacements reported findings similar to our study.^10^ Penfold and colleagues identified comparable model performance for mortality after hip and knee replacements at 90 and 365 days after surgery, with slightly higher model discrimination at the earlier time point. This finding is expected given that ASA and all three comorbidity scores were derived to predict early postoperative mortality. Penfold and colleagues also concluded that comorbidity scores added little improvement to model performance.

### Strengths and limitations of this study

Mortality is a rare event after primary elective shoulder replacement surgery, and hence the main strength of our study was the large sample size and use of linked national joint registry and hospital data. The data reflected all of the main types of primary shoulder replacement procedures undertaken in different geographical regions in patients of various ages and ethnic and socioeconomic groups, providing a complete picture of shoulder replacement activity across a national healthcare system. Robust internal validation techniques and the use of decision curve analysis ensured that all aspects of model performance were accurately evaluated. By investigating the inclusion of multiple comorbidity scores, the true value of including information on comorbidities was captured.

Despite these strengths, the variables considered for inclusion were limited by the data available and the study was instead focused on identifying whether comorbidity variables (ie, that require linkage to hospital data) are necessary to improve model performance compared with data that are routinely collected by national joint registries. More accurate models for predicting mortality, and especially revision surgery, might require inclusion of other variables, some of which might not be collected by joint registries or hospitals. The more complex models showed increased model optimism, reflecting a degree of uncertainty about the incremental benefit from adding comorbidities, but the data in this study currently represent one of the largest and most complete shoulder replacement registries in the world. Although decision curve analysis provided an additional measure of model performance, an improved understanding of the range of relevant risk thresholds in the specialty is necessary to help improve assessments of net benefit in future research. The models were generated by using data from patients who underwent surgery, meaning that the findings are conditional on patients being considered fit enough to proceed with elective shoulder replacement surgery. Finally, external validation was not performed, but would have been essential only if the specific models were being proposed for predicting risk in individual patients; instead, this study focused on identifying the importance of comorbidity variables.

### Conclusions

Case mix adjustment is an important consideration if National Joint Registry data are used for surgeon and hospital outlier analysis. In this study, we found little additional benefit of including comorbidity measures beyond simpler models (ie, that included age, sex, ASA grade, and main surgical indication) for predicting mortality at 90 and 365 days and long term revision surgery after primary elective shoulder replacement surgery. Therefore, the addition of comorbidity scores from other databases needs to be balanced against the additional burden and logistic complexity of data linkage.

## Supplementary material

10.1136/bmjmed-2024-001283 online supplemental file 1

## Data Availability

Data may be obtained from a third party and are not publicly available.
