# Supplementary material for: Evaluation of comorbidity measures for predicting mortality and revision surgery after elective primary shoulder replacement surgery based on data from the National Joint Registry and Hospital Episode Statistics for England: population based cohort study
Source: BMJ Med. 2025 Aug 10;4(1):e001283. doi: 10.1136/bmjmed-2024-001283 (PMC12336573; doi:10.1136/bmjmed-2024-001283)
Supplement: online supplemental file 1 [file bmjmed-4-1-s001.pdf]

Supplementary material

Forest plots 2
365-day mortality 2
Calibration slope 2
C-index 3
Revision surgery 4
Calibration slope 4
C-index 5

Forest plots

365-day mortality

Calibration slope

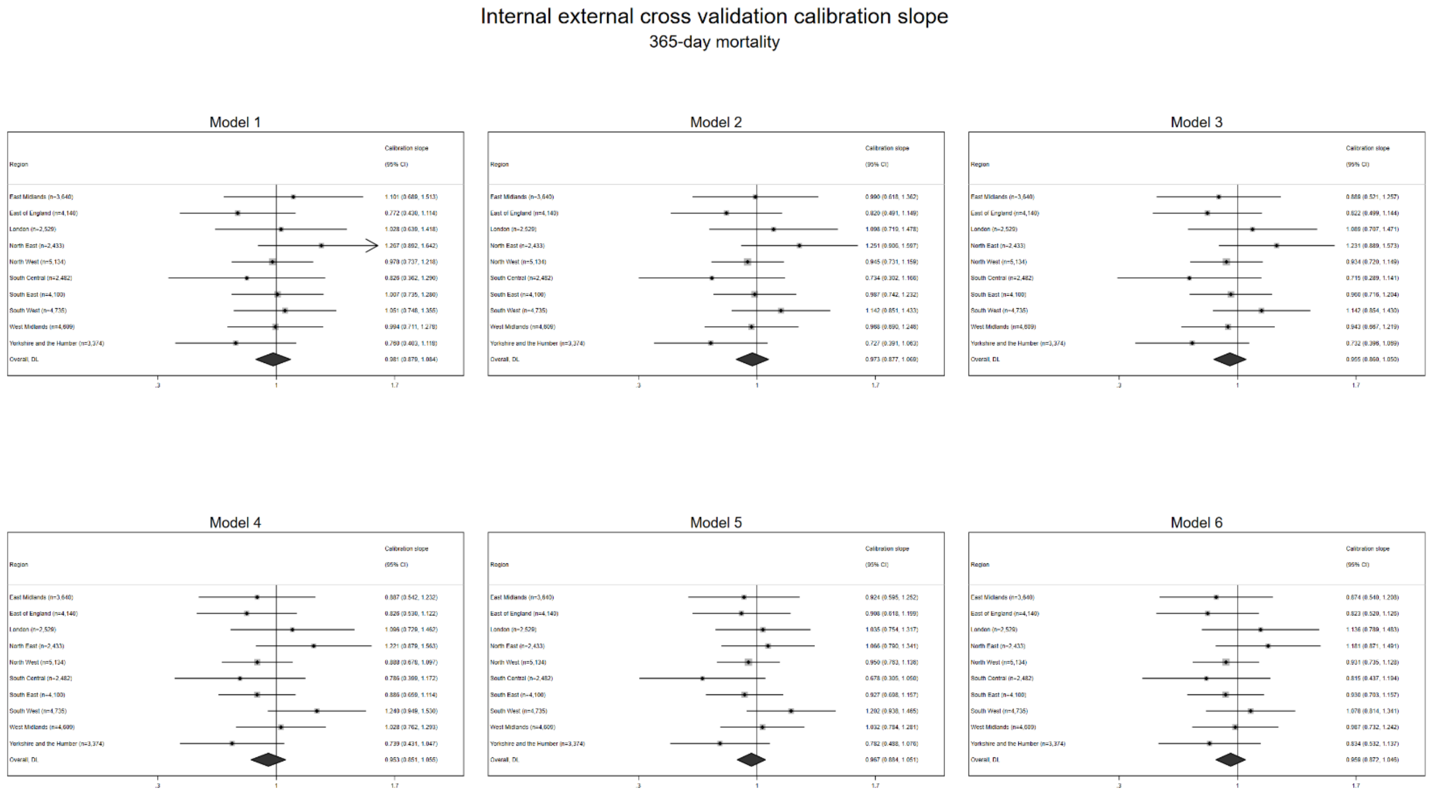

Internal external cross-validation by treatment region for model calibration slope for 365-day mortality. Solid vertical lines indicate perfect calibration slope and calibration-in-the-large. Small black diamonds indicate point estimates, bars indicate 95% CIs, grey squares indicate region weight, large diamonds indicate pooled estimates from the random-effects meta-analysis.

C-index

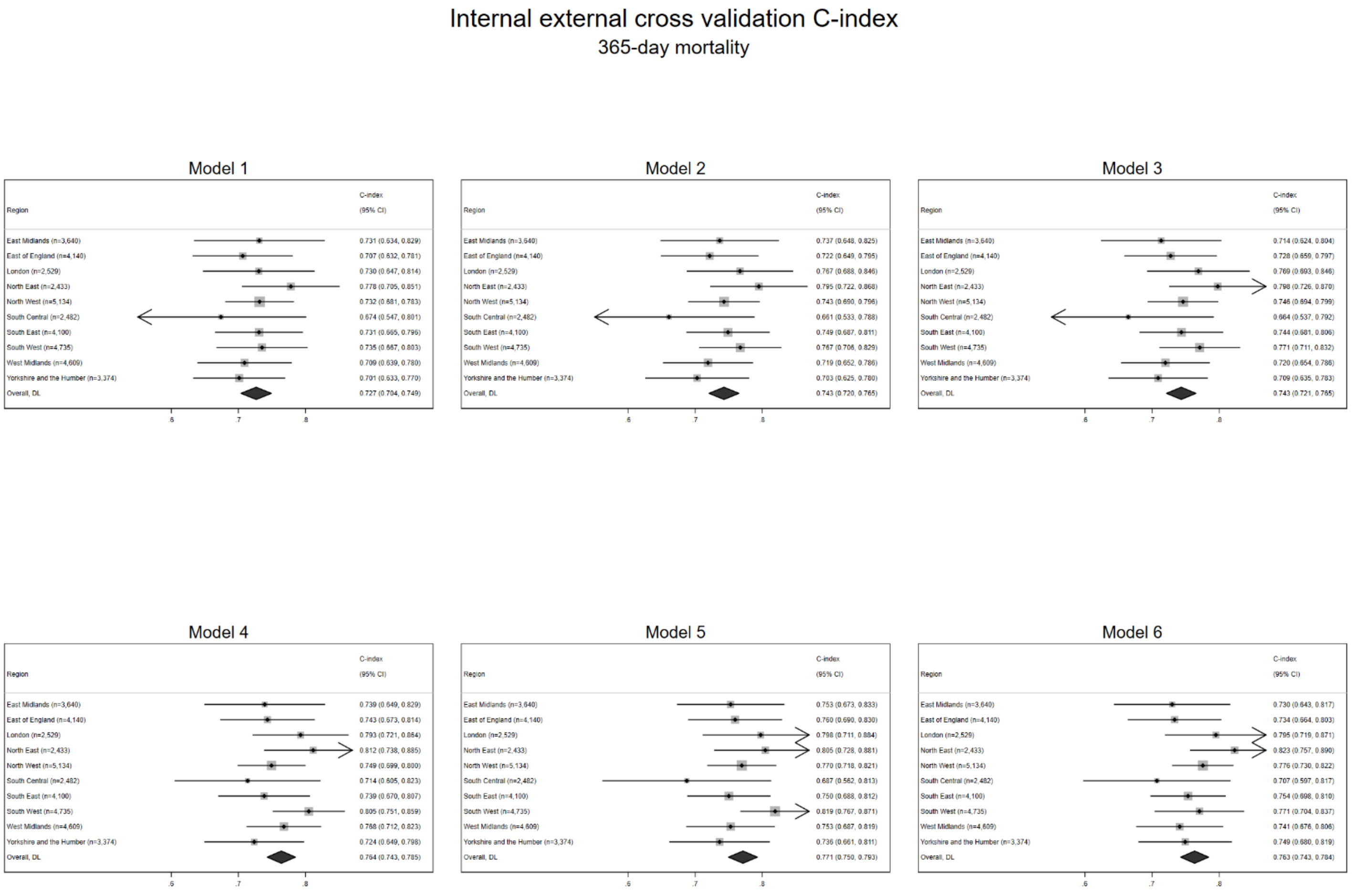

Internal external cross-validation by treatment region for model C-index for 365-day mortality. Solid vertical lines indicate perfect calibration slope and calibration-in-the-large. Small black diamonds indicate point estimates, bars indicate 95% CIs, grey squares indicate region weight, large diamonds indicate pooled estimates from the random-effects meta-analysis.

Revision surgery

Calibration slope

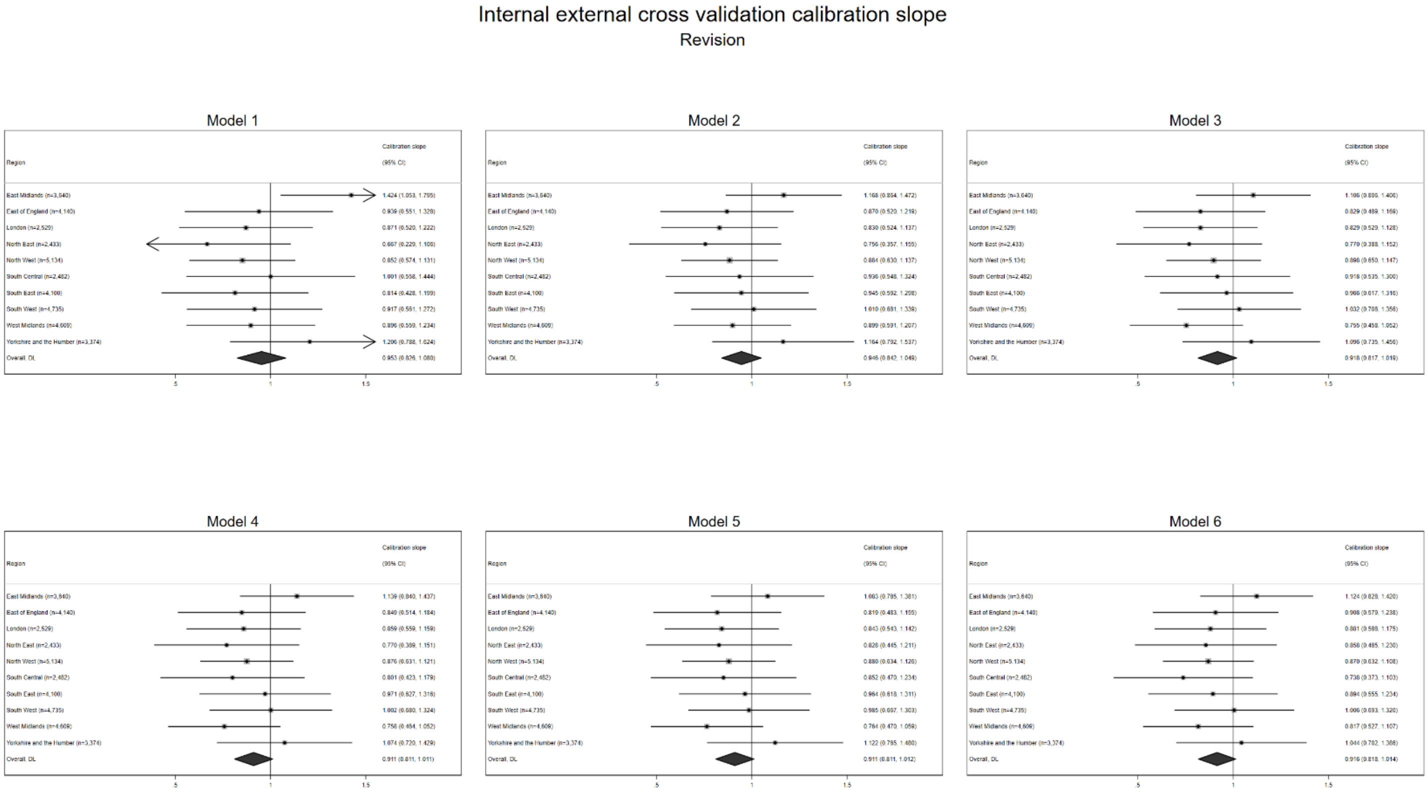

Internal external cross-validation by treatment region for model calibration slope for revision surgery. Solid vertical lines indicate perfect calibration slope and calibration-in-the-large. Small black diamonds indicate point estimates, bars indicate 95% CIs, grey squares indicate region weight, large diamonds indicate pooled estimates from the random-effects meta-analysis.

C-index

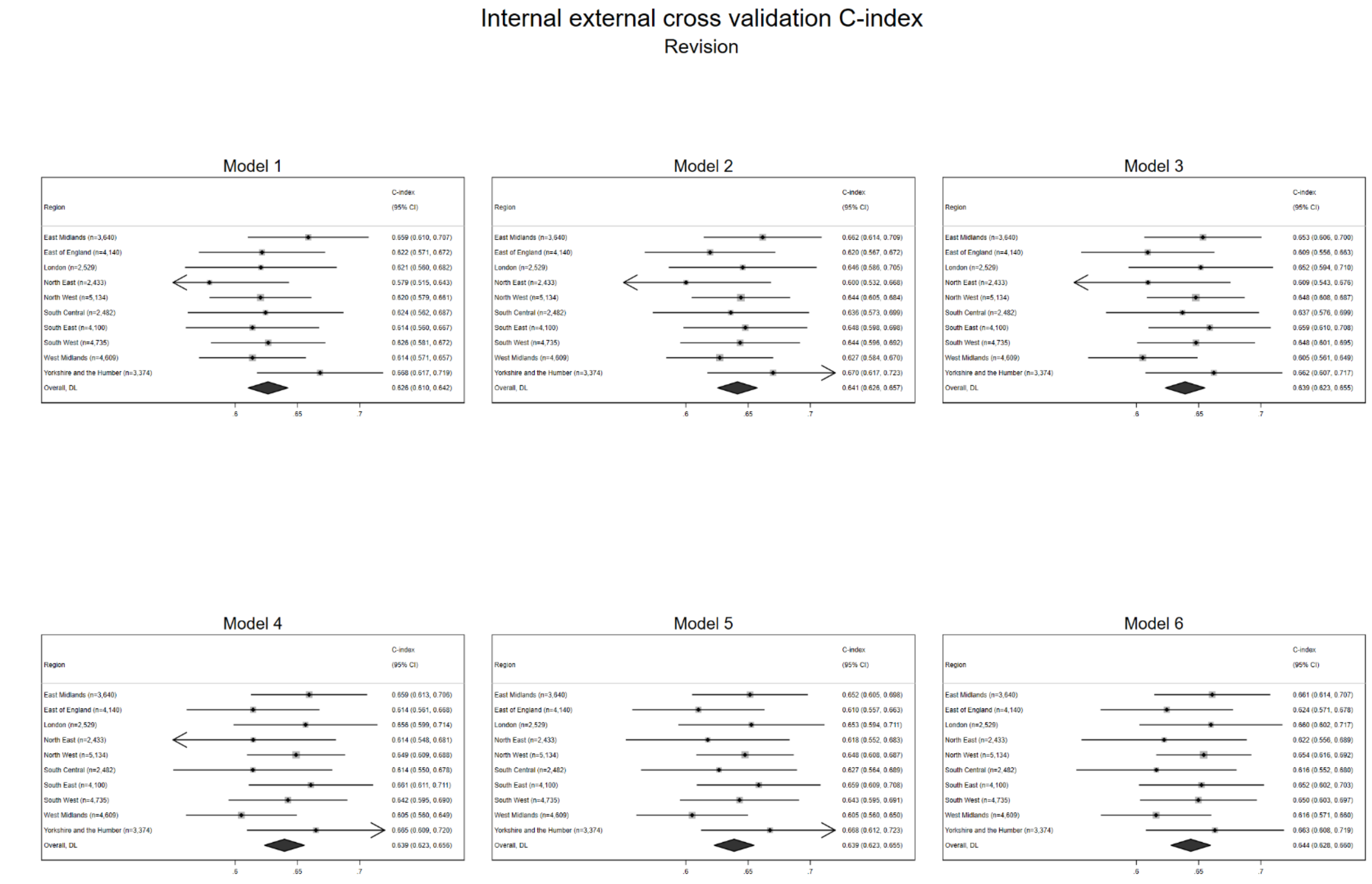

Internal external cross-validation by treatment region for model C-index for revision surgery. Solid vertical lines indicate perfect calibration slope and calibration-in-the-large. Small black diamonds indicate point estimates, bars indicate 95% CIs, grey squares indicate region weight, large diamonds indicate pooled estimates from the random-effects meta-analysis.
