# Supplementary material for: Evaluation of comorbidity measures for predicting mortality and revision surgery after elective primary shoulder replacement surgery based on data from the National Joint Registry and Hospital Episode Statistics for England: population based cohort study
Source: BMJ Med. 2025 Aug 10;4(1):e001283. doi: 10.1136/bmjmed-2024-001283 (PMC12336573; doi:10.1136/bmjmed-2024-001283)
Supplement: Supplementary file 2 [file bmjmed-2024-001283.reviewer_comments.pdf]

PEER REVIEW HISTORY

BMJ Medicine publishes all reviews undertaken for accepted manuscripts. Reviewers are asked to complete a checklist review form and are provided with free text boxes to elaborate on their assessment. These free text comments are reproduced below.

ARTICLE DETAILS

**TITLE (PROVISIONAL)**	An evaluation of comorbidity measures for predicting mortality and revision surgery after elective primary shoulder replacement surgery: population based cohort study using data from the National Joint Registry and Hospital Episode Statistics for England
**AUTHORS**	Valsamis, Epaminondas; Sayers, Adrian; Ma, Jie; Dhiman, Paula; Gwilym, Stephen E; Rees, Jonathan L

VERSION 1 - REVIEW

**REVIEWER NAME**	Sain, Baijaeek
**REVIEWER AFFILIATION**	Ashford and Saint Peter's Hospitals NHS Trust, Trauma & Orthopaedics
**REVIEWER CONFLICT OF INTEREST**	None
**AI DISCLOSURE**	No
**DATE REVIEW RETURNED**	06-Feb-2025

**GENERAL COMMENTS**	Excellent research work. No major revisions required. Minor revision - might it be perhaps possible to add the different modes of primary prosthesis used- anatomical, total, reverse, CTA etc for the primary arthroplasties done in your demographics table. Once revised, can be

**REVIEWER NAME**	Gamarra, Olinda
**REVIEWER AFFILIATION**	Universidad Privada del Este Cde, Alto Parana
**REVIEWER CONFLICT OF INTEREST**	None
**AI DISCLOSURE**	No
**DATE REVIEW RETURNED**	06-Feb-2025

**GENERAL COMMENTS**	No hay cambios sugeridos

**REVIEWER NAME**	Riley, Richard
**REVIEWER AFFILIATION**	University of Birmingham, Institute of Applied Health Research
**REVIEWER CONFLICT OF INTEREST**	I collaborate with author Dhiman
**AI DISCLOSURE**	No
**DATE REVIEW RETURNED**	02-Mar-2025

**GENERAL COMMENTS**	Thank you for the opportunity to review this interesting study. The authors have clearly worked hard to produce this research and it is well written. I have reviewed this from a statistical perspective, and have some comments for improvement.

	1) The objectives of this study are somewhat unclear to me. The paper seems to have 3 related aims, of improving outlier detection for poor performing surgeons and practices; identifying the most important predictors of individual risk; and developing well calibrated and clinically useful prediction models for individual risk. The paper findings are driven by the prediction models; the model-building methodology is very appropriate, but I am not sure that fully links to the desired aim to make sure we properly account for case-mix adjustment when evaluating practices, in terms of what factors to consider. The latter should also consider the causal pathway, and what are causal factors of mortality risk, not just what variables have been selected for inclusion in the prediction model? So I was just left a bit confused.<br>2) Given the strength of the paper is on the actual models, I began to wonder what existing models there are for mortality risk after shoulder surgery, and how these models compare – both in terms of their performance and their including predictors<br>3) With these points in mind, the conclusion that 'Patient variables including age, sex, ASA grade and main surgical indication are sufficient to accurately predict mortality after elective primary shoulder replacement surgery' seems wildly overstated. What is sufficient? I mean, the c-statistic is 0.74, so there is clearly much room for improvement. If another variable was added that moved the c-statistic to 0.90, then surely this would be better still?<br>4) The authors say that adding patient comorbidity scores makes no important impact: perhaps this is the most important part of the paper actually. That is, whether accounting for comorbidities has an impact on individual risk, and thus whether it should be accounted for when comparing practices and surgeons etc. That comes across better in the 'what this study adds' part, so I suggest the title of the paper also reflects this, and also the introduction of the abstract and main paper<br>5) Is mortality the best outcome to consider? I would not expect shoulder surgery to be so worried about mortality outcomes, but rather about shoulder movement/pain. Would we really assess/compared shoulder surgeons on mortality outcomes?<br>6) The methods for the prediction modelling are generally well done, but I have a few queries. Firstly, 'Separate models were developed for each time-point' – this does not make sense as the flexible parametric model actually models all time-points together, and so allows predictions by ANY time-point. So, it's not clear what the authors have done here. Perhaps the different models censor patients at different time points?<br>7) How were the thresholds chosen for the decision curve analysis? This is important, and should be done before the analysis with clinical stakeholders – however, here it seems the authors have just considered all possible thresholds. Can they justify this?<br>8) Calibration plots should have calibration curves added, based on the individual (i.e. non grouped data), to provide the more complete picture of performance, alongside the 10 groups on each plot. For example, using pseudo observations.<br>9) I did not see any justification in the Methods section for the sample size and number of predictor parameters used in the modelling . There are no details on whether (automated) variable selection took place<br>10) Optimism adjusted results are shown, but it is not clear if the final model equations were also adjusted for optimism (penalisation / shrinkage)

	11) The results section should mention what the optimism actually was – it is quite large actually for the more complex models.<br>12) The discussion should mention that the study may not be powered to properly evaluate the added value of comorbidities. That is, the optimism (and thus instability) in the more complex models is much larger than the standard models. This means there is the need for more shrinkage due to the more parameters, which potentially dilutes the effect of the comorbidities. A subtle but important point<br>13) The calibration slope is potentially misleading, as I think (based on my experience) it is being calculated across ALL time points and not the time point of interest. That is, does the slope relate to the calibration plots at ONE particular time point, or based all the survival model across all timepoints? I think it is the latter, which is why we see it is a slope of 1 on apparent performance – as it is should be – but would not necessarily be at one time point of interest.<br>14) The calibration plots: are these after optimism adjustment? Or are these the apparent fit?<br>15) Given the importance of practice-specific performance in this setting (i.e., whether predictions and findings remain the same in each practice), I was surprised there was not more investigation of heterogeneity of model performance across practices (hospitals)<br><br>I hope these comments are constructive and helpful to the authors moving forwards, and I look forward to seeing the revision.

**REVIEWER NAME**	Blake, Helen
**REVIEWER AFFILIATION**	
**REVIEWER CONFLICT OF INTEREST**	None
**AI DISCLOSURE**	No
**DATE REVIEW RETURNED**	03-Apr-2025

**GENERAL COMMENTS**	Thank you for the opportunity to review this interesting manuscript on case-mix adjustment of mortality after elective primary shoulder replacement surgery. I thought the manuscript was relatively short, and would recommend adding to the manuscript, particularly by elaborating on the value of this work and giving more details on methodological choices and results. Specific suggestions follow:<br>● Emphasize why it is important to predict the mortality risk of this procedure (given mortality is, thankfully, relatively low). Why not predict the risk of post-operative complications or another outcome measure?<br>● How did public and patient involvement (PPI) contribute to the research? What impacts did this have on the study?<br>● The authors chose a cubic spline rather than an alternative non-linear representation (e.g. simply adding a quadratic term). Please can you add details to justify this decision to readers?<br>● The amount of missing data was fairly low, and the analysis was done on complete records. I would appreciate more detail on the assumptions underlying this decision, and consideration of whether other missing data methods are appropriate and/or feasible in this case.<br>● I suspect most of the audience is not familiar with risk-adjustment methodology – please elaborate on optimism-adjusting, and the concept of good performance (i.e. should they expect 100% performance or not).<br>● The methods should also provide more detail on model

	development and performance, for example:<br>○ Backwards versus forwards modelling?<br>○ Does the order in which variables are added to the model make a difference?<br>○ How was the number of bootstraps was chosen?<br>○ How did the authors decide what "sufficient" performance was?<br>○ Justification for which variables to use?<br>● In the results section, it is not clear what the final models were, and whether the same variables were chosen for short-term and 1 year mortality (though I note the models are briefly described at the end of the discussion section).<br>● How do the final chosen models compare to the other risk models that were mentioned in the introduction?<br>● Regarding the discussion on the intended purpose of the final models (i.e. case-mix adjustment), please explicitly state whether or not they should be used for individual risk prediction (as clinicians will be tempted to do this anyway, even though the authors have said the models are not explicitly designed for prediction).<br><br>— Other minor comments include:<br>● Please provide more context on primary shoulder replacement surgery for non-expert audience. For example, how common is the procedure? Does 'primary' mean only one shoulder is being operated on? Or this is the first procedure for the patient?<br>● In the results, "Patients who died at both time points" sounds like people died twice. I suggest editing to something along the following lines: 'At both time points, people who died were on average more likely…'.<br>● It is not clear in Table 1 which IMD category maps to the most deprived decile or least deprived.<br>● There are some small numbers in Table 1 – should these be suppressed to protect patient anonymity? For example, by combining ASA grades 1 and 2?<br>● It is not clear in Figure 1 what the red lines are.<br>● The net benefit lines in Figure 2 are hard to distinguish for a colourblind reader.

VERSION 1 – AUTHOR RESPONSE

We would like to thank the reviewers for their constructive comments which have improved the quality and readability of the manuscript. Please let us know if there are any other changes you feel would further improve the manuscript. Below, we address all comments in order, including our responses to these in red text.

Reviewer: 1
Dr. Baijaeek Sain, Ashford and Saint Peter's Hospitals NHS Trust, AMRI Hospitals
Comments to the Author
Excellent research work. No major revisions required. Minor revision- might it be perhaps possible to add the different modes of primary prosthesis used- anatomical, total, reverse, CTA etc for the primary arthroplasties done in your demographics table. Once revised, can be

Response #1: Thank you for your positive comments about our paper. We have now added the proportions for the different types of shoulder replacements in our study cohort in Table 1.

Reviewer: 2
Dr. Olinda Gamarra, Universidad Privada del Este Cde, Universidad de la Integracion de las Americas
Comments to the Author
No hay cambios sugeridos

Response #2: Muchas gracias por sus comentarios positivos.

Reviewer: 3
Prof. Richard Riley, University of Birmingham
Comments to the Author
Thank you for the opportunity to review this interesting study. The authors have clearly worked hard to produce this research and it is well written. I have reviewed this from a statistical perspective, and have some comments for improvement.

1) The objectives of this study are somewhat unclear to me. The paper seems to have 3 related aims, of improving outlier detection for poor performing surgeons and practices; identifying the most important predictors of individual risk; and developing well calibrated and clinically useful prediction models for individual risk. The paper findings are driven by the prediction models; the model-building methodology is very appropriate, but I am not sure that fully links to the desired aim to make sure we properly account for case-mix adjustment when evaluating practices, in terms of what factors to consider. The latter should also consider the causal pathway, and what are causal factors of mortality risk, not just what variables have been selected for inclusion in the prediction model? So I was just left a bit confused.

Response #3: Thank you for highlighting this. The aim of our study was to assess whether the addition of comorbidity measures (through additional linkage of registry data to hospital records) is necessary when undertaking case-mix adjustment for outlier analysis. Most national joint registries collect specific patient and surgical characteristics, but they do not collect comorbidity information. Therefore, the model parameters we included were heavily influenced by the data that is routinely collected by national joint registries (which is mentioned in the limitations, and which we now also clarify in the 'statistical analysis' section of the methods). We did not develop models with the intent for them to be used for individual risk prediction, and registries use different outlier detection methods in different registries. We appreciate that this focus was unclear in our original manuscript, and we have made several changes throughout the paper, including the abstract and title to improve this, also considering your points in your comment #4.

2) Given the strength of the paper is on the actual models, I began to wonder what existing models there are for mortality risk after shoulder surgery, and how these models compare – both in terms of their performance and their including predictors

Response #4: Previous research has focused on identifying associations of certain variables with the risk of mortality after shoulder replacement, and we are unaware of an existing prediction model for this. However, we have hopefully clarified that our aim was not to develop prediction models for individualised prediction, but to use the models to evaluate the importance of comorbidity measures

compared to the variables available in joint registries.

3) With these points in mind, the conclusion that ‘Patient variables including age, sex, ASA grade and main surgical indication are sufficient to accurately predict mortality after elective primary shoulder replacement surgery’ seems wildly overstated. What is sufficient? I mean, the c-statistic is 0.74, so there is clearly much room for improvement. If another variable was added that moved the c-statistic to 0.90, then surely this would be better still?

Response #5: Thank you for highlighting this. We have changed this wording to reflect the fact that adding comorbidities to simpler models offers no improvement, and do not write that any model is ‘sufficient’. Additionally, in the first paragraph of our discussion section we have amended the term ‘sufficient’ to ‘good’ for mortality and ‘moderate’ for revision, as we agree that there is certainly room for improvement here.

4) The authors say that adding patient comorbidity scores makes no important impact: perhaps this is the most important part of the paper actually. That is, whether accounting for comorbidities has an impact on individual risk, and thus whether it should be accounted for when comparing practices and surgeons etc. That comes across better in the ‘what this study adds’ part, so I suggest the title of the paper also reflects this, and also the introduction of the abstract and main paper

Response #6: Thank you for this suggestion. As per our response #3 earlier, we have changed the focus of our manuscript, including the title and abstract, to reflect this.

5) Is mortality the best outcome to consider? I would not expect shoulder surgery to be so worried about mortality outcomes, but rather about shoulder movement/pain. Would we really assess/compared shoulder surgeons on mortality outcomes?

Response #7: Currently, the National Joint Registry identifies outlier surgeons and hospitals for hip and knee replacements based on revision surgery and mortality outcomes, though this has not yet been introduced for shoulder replacements- and this study will hopefully inform this. The rate of serious adverse events, including mortality after shoulder replacement, are higher than originally thought (https://www.bmj.com/content/364/bmj.l298) and therefore mortality is an important outcome to consider. We have clarified this in the introduction but have now also added the second important outcome of revision (redo surgery) which we were originally planning to report separately. However, following both your comment and reviewer #4’s similar comment, we have decided to combine the two outcomes in this paper to provide a more complete picture of any added value of comorbidity measures.

6) The methods for the prediction modelling are generally well done, but I have a few queries. Firstly, ‘Separate models were developed for each time-point’ – this does not make sense as the flexible parametric model actually models all time-points together, and so allows predictions by ANY time-point. So, it’s not clear what the authors have done here. Perhaps the different models censor patients at different time points?

Response #8: Thank you, we have amended the text to clarify that for each mortality model, patients were censored at the two different time-points (90 and 365 days). Investigating mortality at 90 days and at 365 days is common practice in arthroplasty research (as has been previously done for hip and knee replacements) and informed our decision to develop two separate models for mortality.

7) How were the thresholds chosen for the decision curve analysis? This is important, and should be done before the analysis with clinical stakeholders – however, here it seems the authors have just considered all possible thresholds. Can they justify this?

Response #9: Our intention was to use decision curve analysis as another metric (in addition to discrimination and calibration), to evaluate model performance and thus the value of comorbidity measures. As our focus was not to develop models for individualised risk prediction to be used in clinical practice, we did not establish a particular threshold, but feel that decision curve analysis provides useful additional information when comparing models with different variables. We were interested in how models performed across all thresholds as we are investigating their comparative value. We have now clarified this in the methods section.

8) Calibration plots should have calibration curves added, based on the individual (i.e. non grouped data), to provide the more complete picture of performance, alongside the 10 groups on each plot. For example, using pseudo observations.

Response #10: We have added lowess smoothers using the pmcalplot package. However, for mortality (90-day in particular) we suspect that due to a very low event rate, the smoother is not smooth, and we have been unable to resolve this. We have contacted the author of the package to see if this can be addressed. As they stand, we think that it may be best not to include the smoothers on these plots, but are happy to be led by your opinion on whether these plots are worth including given the above.

9) I did not see any justification in the Methods section for the sample size and number of predictor parameters used in the modelling . There are no details on whether (automated) variable selection took place

Response #11: As we did not intend to develop models for individualised risk prediction for use in clinical practice, we did not undertake a formal sample size calculation. Further, we used readily collected registry data and so our sample size was informed by the available data for our given time period. No additional variable selection took place. We have now added these clarifications in the methods section.

10) Optimism adjusted results are shown, but it is not clear if the final model equations were also adjusted for optimism (penalisation / shrinkage)

Response #12: We did not adjust the final model equations for optimism as we do not intend for the models to be used for individualised risk prediction in clinical practice (and so do not report the model

equations).

11) The results section should mention what the optimism actually was – it is quite large actually for the more complex models.

Response #13: We have added to the results section under 'Models' that the optimism was larger for the shorter follow-up period and for the more complex mortality models. We feel that listing the optimism for each metric and for each time period may impair readability in the manuscript but this can added if needed.

12) The discussion should mention that the study may not be powered to properly evaluate the added value of comorbidities. That is, the optimism (and thus instability) in the more complex models is much larger than the standard models. This means there is the need for more shrinkage due to the more parameters, which potentially dilutes the effect of the comorbidities. A subtle but important point

Response #14: Thank you for highlighting this point. We have added this to the limitations section.

13) The calibration slope is potentially misleading, as I think (based on my experience) it is being calculated across ALL time points and not the time point of interest. That is, does the slope relate to the calibration plots at ONE particular time point, or based all the survival model across all timepoints? I think it is the latter, which is why we see it is a slope of 1 on apparent performance – as it is should be – but would not necessarily be at one time point of interest.

Response #15: Yes you are correct. We have added some text to clarify that this is the case in the methods section under 'statistical analysis'.

14) The calibration plots: are these after optimism adjustment? Or are these the apparent fit?

Response #16: These are calibration plots of the apparent fit (as are the decision curves). We have now clarified this in the plots and text.

15) Given the importance of practice-specific performance in this setting (i.e., whether predictions and findings remain the same in each practice), I was surprised there was not more investigation of heterogeneity of model performance across practices (hospitals)

Response #17: Thank you for the suggestion. We have now undertaken internal external cross-validation based on geographical region of treatment and added this to the methods and results section (Figures 5, 6).

I hope these comments are constructive and helpful to the authors moving forwards, and I look forward to seeing the revision.

Reviewer: 4
Dr. Helen Blake
Comments to the Author
— Thank you for the opportunity to review this interesting manuscript on case-mix adjustment of mortality after elective primary shoulder replacement surgery. I thought the manuscript was relatively short, and would recommend adding to the manuscript, particularly by elaborating on the value of this work and giving more details on methodological choices and results. Specific suggestions follow:
● Emphasize why it is important to predict the mortality risk of this procedure (given mortality is, thankfully, relatively low). Why not predict the risk of post-operative complications or another outcome measure?

Response #18: Thank you for your point. Please see our response #7 (to reviewer #3) above, that addresses this point. We have now added a further outcome, revision surgery (redo surgery), to the paper.

● How did public and patient involvement (PPI) contribute to the research? What impacts did this have on the study?

Response #19: Patients and the public were not directly involved in this study. However, the study topic has been identified as important through a James Lind Alliance Priority Setting Partnership and endorsed by the patient network of the National Joint Registry.

● The authors chose a cubic spline rather than an alternative non-linear representation (e.g. simply adding a quadratic term). Please can you add details to justify this decision to readers?

Response #20: Restricted cubic splines are commonly used in survival analysis because of their flexibility and interpretability, as well as being able to capture complex non-linear relationships and ensure smoothness. However, this is not to say that other non-linear specifications would not be suitable. We have added a sentence under 'statistical analysis' in the methods section to explain this to readers. We are concerned that adding further technical details may impair readability for the intended audience.

● The amount of missing data was fairly low, and the analysis was done on complete records. I would appreciate more detail on the assumptions underlying this decision, and consideration of whether other missing data methods are appropriate and/or feasible in this case.

Response #21: The amount of missing data was very low at 1%. Regardless of the mechanism of missingness of the data, it is highly unlikely for this have any effect at all on our results. Therefore, we have followed the accepted rule of thumb in carrying out a complete case analysis when the proportion of missing data is under 5%. We have now added the below references to that section of the paper.

Jakobsen, J.C., Gluud, C., Wetterslev, J. *et al.* When and how should multiple imputation be used for handling missing data in randomised clinical trials – a practical guide with flowcharts. *BMC Med Res Methodol* **17**, 162 (2017). https://doi.org/10.1186/s12874-017-0442-1

Graham, J. W. (2009). Missing Data Analysis: Making It Work in the Real World. Annual Review of Psychology, 60, 549576.
http://dx.doi.org/10.1146/annurev.psych.58.110405.085530

● I suspect most of the audience is not familiar with risk-adjustment methodology – please elaborate on optimism-adjusting, and the concept of good performance (i.e. should they expect 100% performance or not).

Response #22: Thank you for the suggestion. We have added a sentence on model optimism in the methods section, and also added further detail about the C index and calibration slope values in the results section under 'models'.

● The methods should also provide more detail on model development and performance, for example:
○ Backwards versus forwards modelling?
○ Does the order in which variables are added to the model make a difference?
○ How was the number of bootstraps was chosen?
○ How did the authors decide what "sufficient" performance was?
○ Justification for which variables to use?

Response #23: We have now clarified that no additional variable selection techniques were used in the 'statistical analysis' section of the methods. The number of bootstraps chosen was arbitrary, but 200 is more than some recent recommendations have suggested is necessary (https://www.bmj.com/content/bmj/386/bmj-2023-078276.full.pdf). We have altered the wording of 'sufficient' to 'good'/'moderate' as we agree it was not justifiable, and we have avoided this comment elsewhere in the manuscript. Please see our response #3 to Reviewer #3 on the point about choice of variables- we have also added a sentence at the start of the 'statistical analysis' section of the methods to clarify this.

● In the results section, it is not clear what the final models were, and whether the same variables were chosen for short-term and 1 year mortality (though I note the models are briefly described at the end of the discussion section).

Response #24: The variables for each model are listed in the model description column in Table 2 as well as in the captions for each figure- indeed they were the same variables for both time-points of mortality. We have not reported the model equations as we do not intend for them to be used in clinical practice- we were interested in the additional value of comorbidity variables (please see our responses to reviewer #3 on this point).

● How do the final chosen models compare to the other risk models that were mentioned in the introduction?

Response #25: Please see our response #4 to reviewer #3 on this point. In the introduction we

highlight the importance of case-mix adjustment by joint registries but to not refer to any other models as such.

● Regarding the discussion on the intended purpose of the final models (i.e. case-mix adjustment), please explicitly state whether or not they should be used for individual risk prediction (as clinicians will be tempted to do this anyway, even though the authors have said the models are not explicitly designed for prediction).

Response #26: Thank you for highlighting this. This was not the intention of our study as we have now clarified (please see our response #3 to reviewer #3 on this point). Additionally, we are not publishing the model equations in the study, so they cannot be used for individual risk prediction.

— Other minor comments include:

● Please provide more context on primary shoulder replacement surgery for non-expert audience. For example, how common is the procedure? Does ‘primary’ mean only one shoulder is being operated on? Or this is the first procedure for the patient?

Response #27: We have added a sentence in the introduction to give readers an understanding of the frequency of the surgery and have now clarified in the methods section that primary means the first procedure.

● In the results, “Patients who died at both time points” sounds like people died twice. I suggest editing to something along the following lines: ‘At both time points, people who died were on average more likely…’.

Response #28: Thank you for this suggestion, we have reworded this sentence.

● It is not clear in Table 1 which IMD category maps to the most deprived decile or least deprived.

Response #29: We have now added “most deprived” and “least deprived” to categories 1 and 10, respectively, in the table to clarify this.

● There are some small numbers in Table 1 – should these be suppressed to protect patient anonymity? For example, by combining ASA grades 1 and 2?

Response #30: As these numbers are reported on a national level, there is no requirement for suppression of small numbers as per NHS Digital guidance (https://digital.nhs.uk/data-and-information/data-tools-and-services/data-services/hospital-episode-statistics/disclosure-control-methodology-for-hospital-episode-statistics-and-emergency-care-data-set).

● It is not clear in Figure 1 what the red lines are.

Response #31: Thank you for highlighting this. We have added to the caption of the calibration plot figures that this is the spike plot that shows the distribution of events and non-events.

● The net benefit lines in Figure 2 are hard to distinguish for a colourblind reader.

Response #32: We agree that this is a potential problem. However, given the large number of models (lines), using dot/dash combinations is likely to result in an entirely unreadable graph. We are happy to be guided by the journal's copy-editing policies for this.

VERSION 2 – REVIEW

**REVIEWER NAME**	Riley, Richard
**REVIEWER AFFILIATION**	University of Birmingham, Institute of Applied Health Research
**REVIEWER CONFLICT OF INTEREST**	I work with Paula Dhiman on some methodology projects
**AI DISCLOSURE**	No
**DATE REVIEW RETURNED**	19-May-2025

**GENERAL COMMENTS**	I thank the authors for their revision and response to my previous comments. I found these generally appropriate, though it would have been helpful (quicker) to state explicitly what text was changed / added in the revision for each point. It is great to see the outcome of 'revision' added to the paper, rather than publishing that separately, as this provides a much more complete picture than only focusing on mortality. Though the paper is improved, there are still some tensions and areas for improvement, which I will try to articulate below.<br><br>In terms of the smoother for the calibration plot, perhaps you need to reduce the number of points being used on the smoother, to make it more smooth? I agree it looks strange as it stands, for some of the mortality graphs, but I would be loath to just remove it (as the fit looks sensible), as it is important. Try changing the default smoother options.<br><br>Remaining comments – these often relate to the tension of the models not being adjusted for optimism or the results focusing on the apparent performance or not comparing the models. It's not easy to articulate some aspects, but I will do my best, and I hope this is constructive.<br><br>1) The authors are focusing on measures of calibration, discrimination and net benefit …. all measures linked to individual risk prediction … and yet their response to me suggests they do not want to consider (in detail) optimism or overfitting issues still, which would lead to too large values of the c-stat and net benefit, and make calibration look too good, in the development dataset. When I asked about this, the authors say 'We did not adjust the final model equations for optimism as we do not intend for the models to be used for individualised risk prediction in clinical practice (and so do

not report the model equations).' – ok, that's fine, but you are using the models to make inferences about whether certain variables add predictive information, so the potential for optimism is still there, and so the comparisons should account for this, in my opinion.
In terms of adding this optimism, they seem to have done this in the paper at times, but in their response suggest otherwise. They say 'We have added to the results section under 'Models' that the optimism was larger for the shorter follow-up period and for the more complex mortality models. We feel that listing the optimism for each metric and for each time period may impair readability in the manuscript but this can added if needed.' – I still do recommend this is done, and optimism-adjusted performance is the focus (and the difference in models after adding predictors, after adjusting for optimism). For the same reason, I remain surprised the sample size was not considered in advance, and – despite the authors saying they have added a discussion about sample size to the methods section – I cannot see anything about this. Indeed, in their response the authors say 'As we did not intend to develop models for individualised risk prediction for use in clinical practice, we did not undertake a formal sample size calculation. Further, we used readily collected registry data and so our sample size was informed by the available data for our given time period.' – this seems to suggest they did not need a sample size calculation, but they don't argue this in the methods. Further, later they say 'The increased model optimism in the more complex models means that the study may have not been powered to fully evaluate the importance of comorbidities, although the data in this study currently represents one of the largest and most complete shoulder replacement registries in the world'- so there is some confusion or inconsistency here. Indeed, the optimism is quite large based on the adjusted cal slopes of 0.8 for the most complex models, so sample size is relevant.

2) Note that - even though the authors say that they didn't add the optimism, when I look through the results (e.g. the abstract), they do report the optimism adjusted results. So I am now a bit confused. E.g. they say 'Optimism-adjusted performance of models including age, sex, American Society of Anaesthesiologists (ASA) grade and main surgical indication had a …'
3) I am pleased to see the addition of the cross validation by region. Please explain how you fitted the random effects meta-analysis though (i.e. what estimation method for this model and how were CIs derived, e.g. using the Hartung-Knapp approach?). Note that it does not make sense to consider calibration in the omitted study, if you don't include some optimism adjusted for the fitted models. That is, we would never apply the models without shrinkage, so the calibration performance in the omitted region is irrelevant unless the model is adjusted for overfitting first.
4) Figures 5 and 6 provide the IECV results, but this is just for one model – the whole point is to examine models with and without comorbidities, so IECV is not useful unless you do this. Indeed, the model with comorbidities is not even presented here?
5) The abstract says the models were well calibrated, but is this just based on apparent performance? How can this conclusion be made, if so (as good apparent calibration expected). Indeed, the same issue occurs in the main results section: 'Calibration plots of the models' apparent fit for both mortality (at 90 and 365 days) and revision surgery (at 5 years)at both time-points indicate acceptable calibration for all models (Figures 1,2).' – the apparent plots have no merit for such strong conclusions about calibration if there is the

	potential for optimism and overfitting. Better to focus on the hold out regions from the IECV approach (after some shrinkage, see above).<br>6) The abstract says the models demonstrated net benefit across all risk thresholds – but if the models are not to be used for individual risk prediction, what is the point on this statement? Moreover, shouldn't it be focused on whether comorbidities changed (improve) the net benefit? There remains tension in the paper about what the purpose of the fitted models is, and it would be better to ensure that the focus is about the comparison, of whether commodities *add* predictive performance, and not about the net benefit of particular models themselves. Indeed, the authors say this themselves in the methods: they 'were interested in the relative predictive performance of each model when assessed through decision curve analysis'<br>7) The authors say 'No additional variable selection techniques were used' – better to say that 'no variable selection techniques were used during the modelling itself'? To stress that chosen variables are forced in?<br>8) Specific details on how bootstrapping was used to calculate the optimism are needed. How were the CIs for the optimism-adjusted performance estimates obtained? Did these account for the uncertainty in the optimism estimate itself?<br>9) The authors say that there is heterogeneity in the IECV meta-analysis, but the plots in Fig 5 and 6 suggest homogenous results – CIs often overlap considerably. How is their heterogeneity statement quantified? What is tau squared esimtate?<br>10) Figs 1 2 3 and 4 are (I think?) apparent performance, but I can't see this labelled for the reader<br>11) Lastly, I ended up wondering whether the main conclusions are justified. The optimism adjusted c-stat is higher for the more complex models … e.g. moving c stat from 0.74 to 0.77 for mortality , and 0.64 to 0.66 for revision. This might seem small, but in my experience it is not easy to improve the c-stat. What is the optimism adjusted improvement in net benefit? If there is some improvement, then can we really conclude that 'This study found no additional benefit of including comorbidity measures beyond simpler models'<br><br>Sorry to have some many further comments at this stage.

VERSION 2 – AUTHOR RESPONSE

We would like to thank the reviewers for their constructive comments which have improved the quality and readability of the manuscript. Please let us know if there are any other changes you feel would further improve the manuscript. Below, we address all comments in order, including our responses to these in red text.

Reviewer: 3
Prof. Richard Riley, University of Birmingham
Comments to the Author
I thank the authors for their revision and response to my previous comments. I found these generally appropriate, though it would have been helpful (quicker) to state explicitly what text was changed / added in the revision for each point. It is great to see the outcome of 'revision' added to the paper, rather than publishing that separately, as this provides a much more complete picture than only focusing on mortality. Though the paper is improved, there are still some tensions and areas for improvement, which I will try to articulate below.

In terms of the smoother for the calibration plot, perhaps you need to reduce the number of points

being used on the smoother, to make it more smooth? I agree it looks strange as it stands, for some of the mortality graphs, but I would be loath to just remove it (as the fit looks sensible), as it is important. Try changing the default smoother options.

We have been in communication with Joie Ensor, the author of the pmcalplot package in Stata that we have been using and have tried to improve the smoothing with him but this has not worked to date. We remain in conversation with him and will attempt to see if it can be improved using splines. If this is not possible by publication, we will keep the original smoother as submitted as we agree that the fit seems reasonable.

Remaining comments – these often relate to the tension of the models not being adjusted for optimism or the results focusing on the apparent performance or not comparing the models. It's not easy to articulate some aspects, but I will do my best, and I hope this is constructive.

1) The authors are focusing on measures of calibration, discrimination and net benefit …. all measures linked to individual risk prediction … and yet their response to me suggests they do not want to consider (in detail) optimism or overfitting issues still, which would lead to too large values of the c-stat and net benefit, and make calibration look too good, in the development dataset. When I asked about this, the authors say 'We did not adjust the final model equations for optimism as we do not intend for the models to be used for individualised risk prediction in clinical practice (and so do not report the model equations).' – ok, that's fine, but you are using the models to make inferences about whether certain variables add predictive information, so the potential for optimism is still there, and so the comparisons should account for this, in my opinion.

In terms of adding this optimism, they seem to have done this in the paper at times, but in their response suggest otherwise. They say 'We have added to the results section under 'Models' that the optimism was larger for the shorter follow-up period and for the more complex mortality models. We feel that listing the optimism for each metric and for each time period may impair readability in the manuscript but this can added if needed.' – I still do recommend this is done, and optimism-adjusted performance is the focus (and the difference in models after adding predictors, after adjusting for optimism). For the same reason, I remain surprised the sample size was not considered in advance, and – despite the authors saying they have added a discussion about sample size to the methods section – I cannot see anything about this. Indeed, in their response the authors say 'As we did not intend to develop models for individualised risk prediction for use in clinical practice, we did not undertake a formal sample size calculation. Further, we used readily collected registry data and so our sample size was informed by the available data for our given time period.' – this seems to suggest they did not need a sample size calculation, but they don't argue this in the methods. Further, later they say 'The increased model optimism in the more complex models means that the study may have not been powered to fully evaluate the importance of comorbidities, although the data in this study currently represents one of the largest and most complete shoulder replacement registries in the world'- so there is some confusion or inconsistency here. Indeed, the optimism is quite large based on the adjusted cal slopes of 0.8 for the most complex models, so sample size is relevant.

We apologise for the confusion. We do agree that sample size is relevant for our study. However, we were limited to the linked data that was available and have amended the text in our methods section to read that "A sample size of convenience was used for the analysis and was informed by the linked NJR/HES data available for our study period". We have reported this over doing an ad hoc sample size calculation for our study, and we have discussed this in our limitation section where we have made a change to the text ("Although the more complex models showed an increased model optimism, reflecting a degree of uncertainty around the incremental benefit from adding comorbidities, the data in this study currently represents one of the largest and most complete shoulder replacement registries in the world.").

As per your recommendation, we have now shrunk the models based on the optimism-adjusted calibration slope (from bootstrapping) and so the calibration plots and DCA plots now reflect the optimism-adjusted model performance. We have included in the methods section that “Shrinkage was applied to the original model coefficients using the optimism-adjusted calibration slope.”

2) Note that - even though the authors say that they didn’t add the optimism, when I look through the results (e.g. the abstract), they do report the optimism adjusted results. So I am now a bit confused. E.g. they say ‘Optimism-adjusted performance of models including age, sex, American Society of Anaesthesiologists (ASA) grade and main surgical indication had a …’

Sorry for the confusion. To be clear, we do report the apparent and optimism-adjusted results, but we did not explicitly report the optimism (the difference between optimism-adjusted performance and apparent performance). However, we have now added a note to caption of Table 2 to clarify this: “The model optimism for each performance metric is represented by the difference between the optimism-adjusted model performance from the apparent model performance.”. We now also report the optimism-adjusted calibration plots and DCA plots after shrinking the models instead of the original plots of the apparent model performance.

3) I am pleased to see the addition of the cross validation by region. Please explain how you fitted the random effects meta-analysis though (i.e. what estimation method for this model and how were CIs derived, e.g. using the Hartung-Knapp approach?). Note that it does not make sense to consider calibration in the omitted study, if you don’t include some optimism adjusted for the fitted models. That is, we would never apply the models without shrinkage, so the calibration performance in the omitted region is irrelevant unless the model is adjusted for overfitting first.

Thank you for the suggestion. We have now clarified that we used the DerSimonian-Laird estimator in the methods section “We used random-effects meta-analysis using the DerSimonian-Laird estimator to calculate the pooled C-index and calibration slope across regions, and forest plots were examined to assess heterogeneity”.

Regarding your point about calibration slope, our understanding of your suggestion is that we undertake bootstrap internal validation for each IECV iteration prior to evaluating the calibration slope on the held-out region. This would be too computationally demanding and is unfortunately not something we can do but we hope that what we report is useful as a way to investigate for heterogeneity across regions of England. However, if you think that it would hence be preferable to remove the reporting of calibration in the IECV and only report the C-index, we can do this instead.

4) Figures 5 and 6 provide the IECV results, but this is just for one model – the whole point is to examine models with and without comorbidities, so IECV is not useful unless you do this. Indeed, the model with comorbidities is not even presented here?

Thank you for your suggestion. We have now undertaken IECV for all 6 models. Due to the large number of plots, we have now included this in the supplementary material. We now report in the results that “There was little heterogeneity in model performance across different geographical regions for all models for each outcome (see supplementary material).”

5) The abstract says the models were well calibrated, but is this just based on apparent performance? How can this conclusion be made, if so (as good apparent calibration expected). Indeed, the same issue occurs in the main results section: ‘Calibration plots of the models’ apparent fit for both mortality (at 90 and 365 days) and revision surgery (at 5 years)at both time-points indicate acceptable calibration for all models (Figures 1,2).’ – the apparent plots have no merit for such strong conclusions

about calibration if there is the potential for optimism and overfitting. Better to focus on the hold out regions from the IECV approach (after some shrinkage, see above).

We have amended the wording in the abstract and main paper to focus on any difference in model performance made by the addition of comorbidity variables, and avoid reporting whether or not performance was good or not. In the abstract we now write: “There was little heterogeneity in model performance across regions of England, and decision curve analysis demonstrated minimal improvement in net benefit when including comorbidity measures.”. We also focus on reporting optimism-adjusted performance instead of apparent performance. In the results section we now write “Calibration plots of the models’ optimism-adjusted fit for both mortality (at 90 and 365 days) and revision surgery (at 5 years) are shown in Figures 1 and 2.”

6) The abstract says the models demonstrated net benefit across all risk thresholds – but if the models are not to be used for individual risk prediction, what is the point on this statement? Moreover, shouldn’t it be focused on whether comorbidities changed (improve) the net benefit? There remains tension in the paper about what the purpose of the fitted models is, and it would be better to ensure that the focus is about the comparison, of whether commodities *add* predictive performance, and not about the net benefit of particular models themselves. Indeed, the authors say this themselves in the methods: they ‘were interested in the relative predictive performance of each model when assessed through decision curve analysis’

As above, we have made changes to focus on optimism-adjusted model performance, and specifically, on any improvement from the addition of comorbidities. In the abstract we now write: “There was little heterogeneity in model performance across regions of England, and decision curve analysis demonstrated minimal improvement in net benefit when including comorbidity measures.”.

7) The authors say ‘No additional variable selection techniques were used’ – better to say that ‘no variable selection techniques were used during the modelling itself’? To stress that chosen variables are forced in?

We have made this change in the text, thank you for the suggestion.

8) Specific details on how bootstrapping was used to calculate the optimism are needed. How were the CIs for the optimism-adjusted performance estimates obtained? Did these account for the uncertainty in the optimism estimate itself?

We have now added to the methods section that we used Harrell's bias correction approach and the CI’s were calculated using the location-shifted bootstrap confidence interval approach described in the following paper which we now reference in the manuscript: https://onlinelibrary.wiley.com/doi/10.1002/sim.9148. We now write in the methods section: . Internal validation with bootstrapping (with 200 bootstrap samples) was used to assess model overfitting; Harrell’s bias correction was used, and confidence intervals calculated using the location-shifted approach [16].”

9) The authors say that there is heterogeneity in the IECV meta-analysis, but the plots in Fig 5 and 6 suggest homogenous results – CIs often overlap considerably. How is their heterogeneity statement quantified? What is tau squared esimtate?

Thank you for your comment. On reflection, we do agree that it the performance is quite homogeneous and have changed the text to reflect this. We now write: “There was little heterogeneity

in model performance across different geographical regions for all models for each outcome (see supplementary material)."

10) Figs 1 2 3 and 4 are (I think?) apparent performance, but I can't see this labelled for the reader

These calibration plots and DCA plots now report the optimism-adjusted performance of the shrunk models and have amended the first sentence of each Figure caption to clarify this (in addition to describing it in the methods section).

11) Lastly, I ended up wondering whether the main conclusions are justified. The optimism adjusted c-stat is higher for the more complex models … e.g. moving c stat from 0.74 to 0.77 for mortality , and 0.64 to 0.66 for revision. This might seem small, but in my experience it is not easy to improve the c-stat. What is the optimism adjusted improvement in net benefit? If there is some improvement, then can we really conclude that 'This study found no additional benefit of including comorbidity measures beyond simpler models'

Thank you for highlighting this point. While we agree that it may be unfair to completely dismiss the small improvements in model performance, we have now clarified our argument in the manuscript that this minimal improvement may not be worth the challenges associated with obtaining these additional variables through linkage with hospital databases. This is due to the additional costs, time, logistical difficulties and inherent uncertainty from the addition of further variables that are inevitably susceptible to coding errors. We now write in the discussion section that "We found that more complex models using more variables, including comorbidity scores, offered minimal improvement in model performance for either outcome. Deriving comorbidity scores can be challenging as it requires linkage of national joint registry data to other hospital datasets- an approach that carries added burden, logistical difficulties and potential coding errors. Given these challenges, routine data linkage for comorbidity variables for predicting mortality at 90 and 365 days or long-term revision surgery after elective primary shoulder replacement surgery is unlikely to be justified."

Concluding, we write: "This study found minimal additional benefit of including comorbidity measures beyond simpler models that included age, sex, ASA grade and main surgical indication for predicting mortality at 90 and 365 days, and long-term revision surgery after primary elective shoulder replacement surgery. Therefore, the addition of comorbidity scores from other databases is unlikely to be justified."

Sorry to have some many further comments at this stage.

Thank you for your constructive comments that have significantly improved the manuscript.

VERSION 3 – REVIEW

**REVIEWER NAME**	Riley, Richard
**REVIEWER AFFILIATION**	University of Birmingham, Institute of Applied Health Research
**REVIEWER CONFLICT OF INTEREST**	None
**AI DISCLOSURE**	No
**DATE REVIEW RETURNED**	23-Jun-2025

**GENERAL COMMENTS**	I thank the authors for clear responses and their improved revision, which they've clearly worked hard on

	A few comments remain:<br>1. In the abstract the model performance for models with comorbidities should be provided so that we can see if the performance is different to the ones without them, which are the ones currently presented I think?<br>2. The details of how bootstrapping was used to derive optimism is still not presented in detail<br>3. After shrinkage of the predictor effects by the optimism adjusted calibration slope (which is mentioned and correct), there are no details about whether the intercept is re-estimated to ensure calibration in the large (whilst fixing the shrunken predictor effects) - this may not have impact on the c statistic results but may impact the calibration and net benefit of the shrunken models, and so is importantly to the potential conclusions<br>4. The authors say that there is minimal improvement in the c statistic but even after optimism adjustment it moves the c stat from 0.74 to 0.77 which is not negligible … some of the decision curves are also amongst the largest for the model with comorbidity (even though they are shrunk the most and so will would look worst in the development dataset if they did not improve performance at all) and so I'm still not confident in the blanket conclusion that comorbidities have no added value<br>5. The authors say that the optimism adjusted calibration slope gets lower and lower from 1 when the model gets more complex and that this indicates a reduction in calibration performance. This is not true - it indicates larger overfitting but after shrinkage we would expect (on average) the calibration to be ok - so refer to greater overfitting and not worse calibration<br>6. Emphasise that IECV do not include adjustment for overfitting in the developed models

VERSION 3 – AUTHOR RESPONSE

We would like to thank the editor and reviewer for their constructive comments which have improved the quality and readability of the manuscript. Please let us know if there are any other changes you feel would further improve the manuscript. Below, we address all comments in order, including our responses to these in red text.

Prof. Richard Riley, University of Birmingham
Comments to the Author
I thank the authors for clear responses and their improved revision, which they've clearly worked hard on
A few comments remain:
1. In the abstract the model performance for models with comorbidities should be provided so that we can see if the performance is different to the ones without them, which are the ones currently presented I think?

Thank you for the suggestion, we have now added the performances of the best performing models that include comorbidity scores in the abstract: "In comparison, the best performing models that included a comorbidity measure had an optimism-adjusted C-index of 0.77 [95% CI 0.73 to 0.81] for 90-day mortality, 0.76 [95% CI 0.74 to 0.78] for 365-day mortality, and 0.65 [95% CI 0.63 to 0.66]."

2. The details of how bootstrapping was used to derive optimism is still not presented in detail

We have now added further details on the bootstrapping approach. The relevant text now reads "Harrell's bias correction was used: in this approach a prediction model is constructed for each

bootstrap sample and its performance is assessed on both the bootstrap sample as well as on the original population. The bootstrap estimate of optimism is then calculated by averaging the difference between bootstrap model performance and original population model performance across all bootstrap samples, and confidence intervals were calculated using the location-shifted approach [16, 17].” We have also included an additional reference for readers.

3. After shrinkage of the predictor effects by the optimism adjusted calibration slope (which is mentioned and correct), there are no details about whether the intercept is re-estimated to ensure calibration in the large (whilst fixing the shrunken predictor effects) - this may not have impact on the c statistic results but may impact the calibration and net benefit of the shrunken models, and so is importantly to the potential conclusions

The intercept was indeed re-estimated and we have now added this to the text: “Shrinkage was applied to the original model coefficients using the optimism-adjusted calibration slope, and the model intercept was re-estimated to ensure calibration in the large.”

4. The authors say that there is minimal improvement in the c statistic but even after optimism adjustment it moves the c stat from 0.74 to 0.77 which is not negligible … some of the decision curves are also amongst the largest for the model with comorbidity (even though they are shrunk the most and so will would look worst in the development dataset if they did not improve performance at all) and so I’m still not confident in the blanket conclusion that comorbidities have no added value

We agree that there is a non-negligible improvement compared to the reference model that includes age, sex and ASA. However, the improvement is much smaller when compared to the model that also includes indication (which is available in registry data) which is the model that we refer to in the main text and abstract (differences in C-index for each outcome are 0.01, 0.02, and 0.01 for 90-day mortality, 365-day mortality and revision, respectively). However, we have now amended the wording of the text across the abstract, summary box and discussion and hope that this is now more balanced:

Abstract conclusion: “Patient comorbidity scores added little improvement beyond simpler models including age, sex, ASA grade and main surgical indication for predicting mortality and revision surgery after elective primary shoulder replacement surgery, and this improvement needs to be balanced against the additional challenges of routine data linkage to obtain those scores.”

What this study adds: “This improvement needs to be balanced against the additional burden and logistic complexity of routine data linkage to derive comorbidity scores.”

How this study might affect research, practice or policy: “When considering data linkage to obtain comorbidity scores, decision-makers should weigh the small performance gain against the practical challenges of implementation.”

Discussion: “We found that more complex models using more variables, including comorbidity scores, offered little improvement in model performance for either outcome. Deriving comorbidity scores can be challenging as it requires linkage of national joint registry data to other hospital datasets- an approach that carries added burden, logistical difficulties and potential coding errors. Given these challenges, the need for routine data linkage to obtain comorbidity variables for predicting mortality at 90 and 365 days or long-term revision surgery after elective primary shoulder replacement surgery needs to be balanced against the small improvement in model performance” and in the final paragraph: “Therefore, the addition of comorbidity scores from other databases needs to be balanced against the additional burden and logistic complexity of data linkage.”

5. The authors say that the optimism adjusted calibration slope gets lower and lower from 1 when the

model gets more complex and that this indicates a reduction in calibration performance. This is not true - it indicates larger overfitting but after shrinkage we would expect (on average) the calibration to be ok - so refer to greater overfitting and not worse calibration

Thank you for highlighting this. We have now amended the text to read: "Among mortality models for both time-points, increasing model complexity was generally associated with a reduction in the optimism-adjusted calibration slope (where 1 would represent perfect alignment of predicted probabilities with observed outcomes), indicating greater overfitting."

6. Emphasise that IECV do not include adjustment for overfitting in the developed models

We have now added the following sentence to the paragraph in the Methods section describing the IECV: "Models were not adjusted for overfitting."

VERSION 4 – REVIEW

**REVIEWER NAME**	Riley, Richard
**REVIEWER AFFILIATION**	University of Birmingham, Institute of Applied Health Research
**REVIEWER CONFLICT OF INTEREST**	I collaborate on some methodology papers with Dhiman
**AI DISCLOSURE**	No
**DATE REVIEW RETURNED**	11-Jul-2025

**GENERAL COMMENTS**	I thank the authors for addressing my latest comments on top of their previous hard work and extensive paper. I look forward to the article being published. My only remaining suggestion is to add a note about the importance of understanding the range of relevant risk thresholds in this field, to help inform net benefit assessments in future research. In particular, in the results section where it says 'For mortality, different models had comparatively greater net benefit at different risk thresholds, meaning there is no one model that could be identified as superior based on decision curve analysis.' – this conclusion is only because the authors are looking at the whole spectrum of possible risk thresholds, and it might be more clear which model is better in a narrower range. This should be emphasised in the discussion.
